# Antibiotic resistance ABCF proteins reset the peptidyl transferase centre of the ribosome to counter translational arrest

**DOI:** 10.1093/nar/gky050

**Published:** 2018-02-05

**Authors:** Victoriia Murina, Marje Kasari, Vasili Hauryliuk, Gemma C Atkinson

**Affiliations:** 1 Department of Molecular Biology, Umeå University, 901 87 Umeå, Sweden; 2 Laboratory for Molecular Infection Medicine Sweden (MIMS), Umeå University, 901 87 Umeå, Sweden; 3 University of Tartu, Institute of Technology, 50411 Tartu, Estonia

## Abstract

Several ATPases in the ATP-binding cassette F (ABCF) family confer resistance to macrolides, lincosamides and streptogramins (MLS) antibiotics. MLS are structurally distinct classes, but inhibit a common target: the peptidyl transferase (PTC) active site of the ribosome. Antibiotic resistance (ARE) ABCFs have recently been shown to operate through direct ribosomal protection, but the mechanistic details of this resistance mechanism are lacking. Using a reconstituted translational system, we dissect the molecular mechanism of *Staphylococcus haemolyticus* VgaA_LC_ and *Enterococcus faecalis* LsaA on the ribosome. We demonstrate that VgaA_LC_ is an NTPase that operates as a molecular machine strictly requiring NTP hydrolysis (not just NTP binding) for antibiotic protection. Moreover, when bound to the ribosome in the NTP-bound form, hydrolytically inactive EQ_2_ ABCF ARE mutants inhibit peptidyl transferase activity, suggesting a direct interaction between the ABCF ARE and the PTC. The likely structural candidate responsible for antibiotic displacement by wild type ABCF AREs, and PTC inhibition by the EQ_2_ mutant, is the extended inter-ABC domain linker region. Deletion of the linker region renders wild type VgaA_LC_ inactive in antibiotic protection and the EQ_2_ mutant inactive in PTC inhibition.

## INTRODUCTION

Approximately half of the antibiotics currently in use for treating bacterial infections inhibit protein synthesis, predominantly by targeting key functional sites of the ribosome (1). To counteract antibiotics, bacteria have developed an array of resistance mechanisms (2). These mechanisms can be broadly classified into two categories. The first strategy is to decrease the intracellular concentration of the active antibiotic. This can be achieved by preventing antibiotic uptake (3), by actively excreting the antibiotic (4) or by inactivating the drug via chemical modification or degradation (5). The second strategy is to render the pathway that is targeted by the antibiotic immune to the drug. This can be achieved by mutation or modification of the antibiotic target (6). Alternatively, a resistance factor can directly interact with the antibiotic target to displace the antibiotic or prevent its binding in the first place, as exemplified by the ribosome protection factors TetO and TetM (7,8). These members of the EF2 family of translational GTPases share the domain structure of elongation factor EF-G (9). Like EF-G, Tet proteins bind to the A-site of the ribosome, where they sterically clash with tetracycline bound to the ribosome, thus effectuating antibiotic dissociation from its target (10,11).

Antibiotic resistance proteins in the ABC (ATP-binding cassette) superfamily of ATPases are well known for conferring resistance through the first of the two strategies, i.e. by reducing the concentration of the drug in the cell by pumping it out (4). However, the crucial component of the classical ABC pump, its transmembrane domain (12,13) is lacking in antibiotic resistance factors that belong to the ABCF subfamily of ABC ATPases (14) (BioRxiv: https://www.biorxiv.org/content/early/2017/11/16/220046). This raises the question of whether resistance is achieved instead via the second strategy described above i.e. via direct protection of the inhibited pathway.

**Figure 1.**
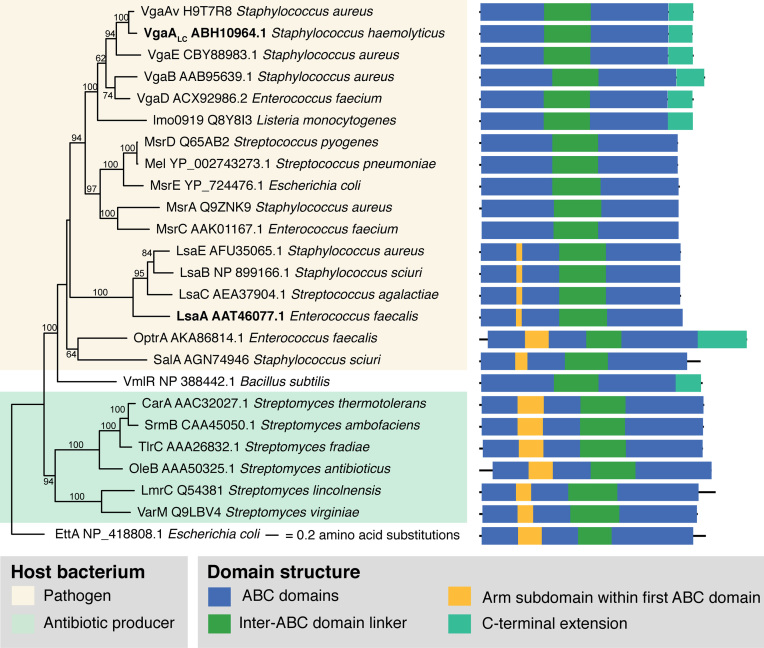
Maximum Likelihood phylogenetic analysis and domain structure of confirmed bacterial ABCF AREs. The tree shows phylogenetic relationships between ABCF AREs from bacterial pathogens (pale yellow), such as *S. haemolyticus* VgaA_LC_ and *E. faecalis* LsaA (highlighted in bold) and bacterial antibiotic producers (pale green). All ABCFs contain two universal ABC domains (blue) separated by an inter-ABC domain linker (green). The first ABC can contain an additional sequence element corresponding to the ‘arm’ subdomain of EttA (32,33) (yellow), and a conserved C-terminal extension can also be present (turquoise). Protein sequences of ABCF AREs presented on the tree were retrieved from the CARD database (37) or from UniProt (36). Numbers on branches show bootstrap support from 100 replicates.

Two archetypal antibiotic resistance (ARE) ABCF factors are VgaA and LsaA, which carry two nucleotide binding domains, separated by a linker region (Figure 1). Staphylococcal VgaA (standing for ‘virginiamycin A-like antibiotic resistance’) is a plasmid-encoded resistance factor described in *Staphylococcus aureus* (15), S*taphylococcus epidermidis* (16) and *Staphylococcus haemolyticus* (17). LsaA (standing for ‘lincosamide and streptogramin A resistance’) is encoded on the chromosome of *Enterococcus faecalis* (18,19). The two terms, ‘virginiamycin A-like antibiotics’ and ‘streptogramin A’ are synonymous and refer to the same chemical group of compounds (20). VgaA and LsaA confer protection against the same antibiotic classes, streptogramin A and lincosamides (so-called LS_A_ phenotype) and pleuromutilins, such as tiamulin, although the relative activity against different antibiotics varies (15–19,21,22). An extreme example is *S. haemolyticus* VgaA_LC_, which has a substrate specificity that is strongly shifted towards lincosamides, such as lincomycin and its derivative, clindamycin (17).

While chemically unrelated, pleuromutilin, streptogramin A and lincosamide antibiotics bind to a common target, the peptidyl transferase centre (PTC) of the large (50S) ribosomal subunit (23–29). The PTC is the catalytic centre of the ribosome, where during each iteration of the elongation cycle the amino acid moiety of an incoming aminoacyl-tRNA to the ribosomal A-site forms a peptide bond with the peptidyl-tRNA in the P-site (30). As the ARE ABCFs confer resistance to antibiotics that are chemically unrelated but share a binding target, it was hypothesized that AREs directly interact with the ribosome to displace the drug (14). A decade later this hypothesis has been experimentally substantiated both for *S. aureus* VgaA and *E. faecalis* LsaA (31). While the molecular mechanism of resistance is still unknown, an inter-ATPase domain linker region that protrudes towards the PTC in the ribosome-binding translation factor ABCF EttA (32,33) is extended in AREs (Supplementary Figure S1). Therefore, it was hypothesized that the linker could directly displace the antibiotic from the PTC (21,34). Indeed, mutations in this region affect the antibiotic specificity of the ARE (21). These initial discoveries have set the stage for dissecting the system biochemically using a reconstituted translational system.

Here, we use antibiotic inhibition of transpeptidation of *S. aureus* 70S ribosomal initiation complexes and rescue by *S. haemolyticus* VgaA_LC_ to probe three aspects of the molecular mechanism of ARE ABCFs. First, we addressed the question of nucleotide substrate specificity. Not all the ABC enzymes are strict ATPases, and certain representatives are, essentially, NTPases that in addition to ATP can utilize GTP, CTP and UTP (35). Second, we clarified the functional role of NTP hydrolysis in the functional cycle of VgaA_LC_. NTPases can operate either as a ‘molecular switch’ where NTP versus NDP binding alters the structural conformation of the enzyme thereby exerting its function, or as a ‘molecular machine’, which uses NTP to NDP hydrolysis to drive a ‘powerstroke’ fueling the performed mechanical work. Both versions are present in the ABC family (12). Finally, we probed structure-function relationships, focusing on the extended alpha-helical linker region connecting the two ATP cassette domains of antibiotic resistance ABCFs (Figure 1).

## MATERIALS AND METHODS

### Phylogenetic analysis

Sequences of previously documented ABCF AREs were retrieved from UniProt (36) and the Comprehensive Antibiotic Resistance Database (CARD) (37). Sequences were aligned with Mafft v7.164b with the l-ins-i and maximum likelihood phylogenetic analysis was carried out with RAxML-HPC v.8 (38) on the CIPRES Science Gateway v3 (39) with 100 bootstrap replicates and the LG model of substitution. Alignment positions with >50% gaps were excluded from the analysis.

### Protein cloning, expression and purification

All cloning was performed by the Protein Expertise Platform at Umeå University. *S. haemolyticus* VgaA_LC_ ORF was PCR-amplified from pRB374 VgaA_LC_ plasmid (17) and sub-cloned to the pET24d expression vector with a C-terminal 6His tag preceded by a single glycine linker. *E. faecalis* LsaA ORF was PCR amplified from pTEX5333 plasmid (18) and sub-cloned into pCA528 vector for 6His-SUMO-tagging (40). ATPase deficient (EQ_2_) mutants were generated by introducing E105Q and E410Q (VgaA_LC_) or E142Q and E452Q (LsaA) point mutations. The linker deletion (ΔL) VgaA_LC_ mutants were generated by preplacing K199-A226 with GSG in ether wt or EQ_2_ VgaA_LC_.

Both LsaA and VgaA_LC_ (wt and mutants) were overexpressed in freshly transformed *Escherichia coli* BL21 DE3 Rosetta (Novagen). An overnight culture in LB supplemented with 50 mg/ml kanamycin and 25 mg/ml chloramphenicol was diluted to 0.06 in the same media, grown at 37°C until an OD_600_ of 0.6–0.7, induced with 1 mM IPTG (final concentration) and grown for 2 h at 30°C. The cells were harvested by centrifugation and resuspended in lysis buffer (VgaA_LC_: 1 M NaCl, 100 mM Tris:HCl pH 7.5, 5 mM imidazole, 10 mM MgCl_2_, 2 mM β-mercaptoethanol, 10% glycerol; LsaA: 0.7 M KCl, 100 mM HEPES pH 7.5, 10 mM imidazole, 0.1% Tween 20, 10 mM MgCl_2_, 2 mM β-mercaptoethanol, 10% glycerol) supplemented with 0.1 mM PMSF, 35 μg/ml lysozyme and 1 U/ml DNase I. Cells were lysed by a high-pressure cell disrupter (Stansted Fluid Power), cell debris removed by centrifugation (35 000 rpm for 40 min) and clarified lysate was taken for protein purification.

As a first step, the supernatants were loaded onto 1 ml HisTRAP HP (GE Healthcare) column equilibrated in buffer A (VgaA_LC_: same as lysis buffer, i.e. 1 M NaCl, 100 mM Tris:HCl pH 7.5, 5 mM imidazole, 10 mM MgCl_2_, 2 mM β-mercaptoethanol, 10% glycerol; LsaA: 0.7 M KCl, 50 mM HEPES pH 7.5, 5 mM MgCl_2_, 10 mM imidazole, 2 mM β-mercaptoethanol). The column was washed with high salt buffer (buffer B) (VgaA_LC_: 2 M NaCl, 100 mM Tris:HCl pH 7.5, 25 mM imidazole, 10 mM MgCl_2_, 2 mM β-mercaptoethanol; LsaA: 2 M KCl, 50 mM HEPES pH 7.5, 20 mM imidazole, 5 mM MgCl_2_, 2 mM β-mercaptoethanol), and the proteins were eluted with a gradient of 0.5 M imidazole buffer (buffer C) (0.7 M KCl, 0.5 M imidazole, 50 mM Tris:HCl pH 7.5, 10 mM MgCl_2_, 2 mM β-mercaptoethanol). The following polishing steps were different for 6His-tagged VgaA_LC_ and 6His-SUMO-tagged LsaA.

In the case of VgaA_LC_, after HisTRAP chromatography the protein was buffer-exchanged on 10 MWCO centricons (Amicon) into low salt buffer (100 mM NaCl, 50 mM Tris:HCl pH 7.5, 5 mM MgCl_2_, 2 mM β-mercaptoethanol) and loaded onto HiPrep Q XL 16/10 (GE Healthcare) column equilibrated in the same buffer. The flow-through fraction was concentrated on 10 MWCO centricons (Amicon) and buffer-exchanged into storage buffer (350 mM KCl, 25 mM HEPES pH 7.5, mM MgCl_2_, 2 mM β-mercaptoethanol, 50% glycerol).

In the case of LsaA, SDS PAGE-pure fractions from HisTRAP chromatography were combined, diluted to a final concentration of KCl of 500 mM in dilution buffer (50 mM HEPES pH 7.5, 5 mM MgCl_2_, 10% glycerol, 2 mM β-mercaptoethanol) and 35 μg of Ulp1 per 1 mg of protein was added. After the 6His-SUMO tag was cut off during buffer exchange to loading buffer (buffer A2) (500 mM KCl, 50 mM HEPES pH 7.5, 5 mM MgCl_2_, 10% glycerol, 2 mM β-mercaptoethanol) on 3 MWCO centricons (Amicon) (1 h at 19°C), the protein was passed though 1 ml HisTRAP HP column (GE Healthcare) pre-equilibrated with LsaA buffer A (see above). Flow-through fractions were collected, diluted to 300 mM KCl (final concentration) in dilution buffer and passed though an anion exchange column (HiPrep Q XL 16/10 20 ml, GE Healthcare) pre-equilibrated with mid-salt buffer (300 mM KCl, 50 mM HEPES pH 7.5, 5 mM MgCl_2_, 2 mM β-mercaptoethanol). The flow-through was collected, concentrated and exchanged into storage buffer (350 mM KCl, 25 mM HEPES pH 7.5, 10 mM MgCl_2_, 2 mM β-mercaptoethanol and 50% glycerol) on 10 MWCO centricons (Amicon).

The purity of protein preparations was assessed by SDS-PAGE and spectrophotometrically (OD_280_/OD_260_ ratio of ≈1.8 for VgaA_LC_ and 1.6–1.8 for LsaA). The proteins were aliquoted and stored at –20°C.

### Preparation of *S. aureus* and *E. faecalis* 70S ribosomes

LB (SH-1000 *S. aureus*) or BHI (OG1RF *E. faecalis*) liquid cultures (12 × 400 ml) were inoculated with an overnight culture to OD_600_ of 0.05–0.06 and grown at 37°C with vigorous shaking. At OD_600_ 2–2.5 (*S. aureus*) or 1.5 (*E. faecalis*), cells were pelleted at 4°C (TLA10.500 rotor, Beckman, 15 min at 5000–8000 rcf), resuspended with either LB (*S. aureus*) or cell opening buffer (20 mM Tris:HCl, 100 mM NH_4_Cl, 15 mM Mg(OAc)_2_, 0.5 mM EDTA, 3 mM mercaptoethanol, pH 7.4), pelleted again in falcon tubes, frozen with liquid nitrogen and stored at –80°C.

*S. aureus* frozen cells (15 g) were opened by cryomilling (Spex Freezer Mill, 8 cycles at 14 fps frequency interspersed with 2 min work-rest intervals). The powder was melted on ice during 3 h before opening the tube in a class II biosafety cabinet, followed by addition of 50 ml of cell opening buffer (100 mM NH_4_Cl, 15 mM Mg(OAc)_2_, 0.5 mM EDTA, 3 mM β-mercaptoethanol, 20 mM Tris:HCl pH 7.5) supplemented with 0.4 mU Turbo DNase (Thermo Fisher Scientific), 0.1 mM PMSF and 35 μg/ml lysozyme and additional incubation on ice for 1 h. *Enterococcus faecalis* frozen cells (15 g) were directly resuspended in cell opening buffer and opened by high-pressure cell disrupter (Stansted Fluid Power) (350 MPa, three passages).

Lysed cells were clarified by centrifugation for 40 min at 40 000 rpm (Ti 45 rotor, Beckman), the supernatant loaded onto sucrose cushions (1.1 M sucrose, 500 mM NH_4_Cl, 15 mM Mg(OAc)_2_, 0.5 mM EDTA, 3 mM β-mercaptoethanol, 20 mM Tris:HCl pH 7.5) and centrifuged for 18–19 h at 28 000 rpm. Ribosomal pellets were dissolved in high salt buffer (500 mM NH_4_Cl, 15 mM Mg(OAc)_2_, 0.5 mM EDTA, 3 mM β-mercaptoethanol, 20 mM Tris:HCl pH 7.5 supplemented with 0.5–1 mM puromycin), incubated for 1 h at 4°C with gentle mixing and pelleted again (8 h at 35 000 rpm or 19 h at 28 000 rpm) through 40 ml sucrose cushions. Resultant ribosomal pellets were combined in 15 ml of overlay buffer (60 mM NH_4_Cl, 15 mM Mg(OAc)_2_, 0.25 mM EDTA, 3 mM β-mercaptoethanol, 20 mM Tris:HCl pH 7.5) and resolved on a 10–40% sucrose gradient in overlay buffer in a zonal rotor (Ti 15, Beckman, 17 h at 21 000 rpm). The peak containing 70S ribosomes was pelleted by centrifugation (20 h at 35 000 rpm), pure 70S dissolved in 1 ml of HEPES:Polymix buffer (20 mM HEPES:KOH pH 7.5, 2 mM DTT, 5 mM Mg(OAc)_2_, 95 mM KCl, 5 mM NH_4_Cl, 0.5 mM CaCl_2_, 8 mM putrescine, 1 mM spermidine) and 70S concentration measured spectrophotometrically (1 OD_260_ = 23 nM of 70S). Ribosomes were aliquoted, frozen in liquid nitrogen and stored at –80°C.

### Preparation of 70S initiation complexes (70S IC)

Initiation complexes were prepared by combining 70S ribosomes (final concentration of 4 μM) with IF2 (2 μM), IF1 (1.5 μM), IF3 (1.5 μM), ^35^S-fMet- tRNA_i_^fMet^ (6 μM), mRNA MF (6 μM, 5′-GGCAAGGAGGUAAAAAUGUUCAAA-3′), 1 mM GTP and 2 mM DTT in 1× HEPES:Polymix buffer. The reaction mix was incubated at 37°C for 30 min, the ICs were pelleted through a sucrose cushion (1.1 M sucrose, HEPES:Polymix buffer 15 mM Mg^2+^ final concentration) at 50 000 rpm during 2 h (TLS-55, Beckman), the pellet was dissolved in 100 μl of HEPES:Polymix buffer (5 mM Mg(OAc)_2_), aliquoted, frozen in liquid nitrogen and stored at –80°C.

### Puromycin reaction

The puromycin reaction was carried out at 37°C in HEPES:Polymix pH 7.5 ^2+^buffer (41). The biochemical system from purified *E. coli* components has been described earlier (42,43). ^35^S-Met-puromycin release was followed using 10% TCA precipitation and centrifugation with scintillation counting of the supernatant (^35^S-Met-puromycin) and pellet (intact ^35^S-fMet-tRNA_i_^fMet^). The percentage of ^35^S-fMethionine released from the 70S IC was calculated by dividing the signal from the supernatant by the sum of signals from supernatant and pellet. The data are presented as geometric means, and error bars represent the range of experimental values.

Detailed experimental protocols, buffer preparations, expression and preparation of initiation factors and Ulp1 protease are found in *SI Materials and Methods*.

## RESULTS

### *S. haemolyticus* VgaA_LC_ ATP-dependently rescues the puromycin reactivity of *S. aureus* initiation complexes inhibited by lincomycin, clindamycin, virginiamycin M1 or tiamulin

**Figure 2.**
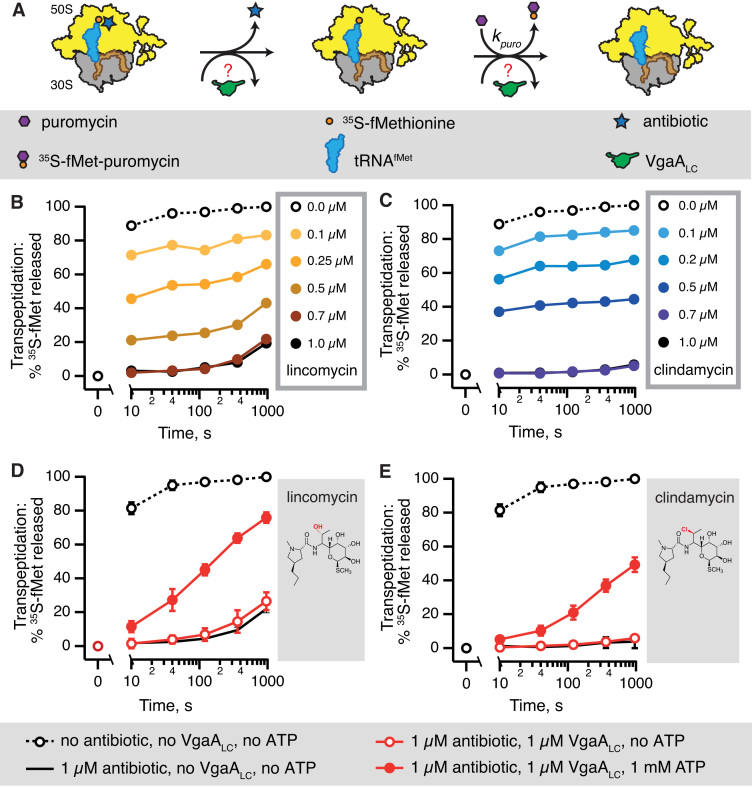
*S. haemolyticus* VgaA_LC_ ATP-dependently counteracts inhibition of puromycin reactivity of *S. aureu*s 70S initiation complexes by lincosamide antibiotics. Puromycin reactivity of *S. aureus* 70S initiation complexes (IC) is used as a test reaction for biochemical studies of *S. haemolyticus* VgaA_LC_ resistance factor protecting against LS_A_ antibiotics targeting the ribosomal peptidyl transferase centre (PTC) (**A**). Increasing concentrations of lincosamides lincomycin (**B**) and its 7(S)-chloro-7-deoxy derivative clindamycin (**C**) abrogate the ^35^S-Methionine release from P-site ^35^S-fMet-tRNA_i_^fMet^ by 1 mM puromycin. At 1 μM both antibiotics saturate the ribosomal complexes and the observed slow puromycin reactivity reflects the kinetics of antibiotic dissociation. In the presence of 1 mM ATP (filled red circles) 1 μM *S. haemolyticus* VgaA_LC_ rescues the puromycin reactivity inhibited by either lincomycin (**D**) or clindamycin (**E**). In the absence of ATP (empty red circles) addition of VgaA_LC_ has no effect. All experiments were performed at 37°C in HEPES:Polymix pH 7.5 buffer, 5 mM Mg^2+^. 0.5 μM *S. aureus* IC(MF) were programmed with synthetic mRNA(MF) encoding Met-Phe dipeptide and *E. coli*^35^S-fMet-tRNA_i_^fMet^ (occupancy 60–80%). ICs were incubated with lincomycin for 5 min to allow equilibrium binding to be achieved prior the addition of puromycin. The data are presented as geometric means, and error bars represent the range of experimental values.

We used the so-called puromycin reaction to assay transpeptidation functionality of the ribosome. The aminonucleoside antibiotic puromycin is a structural mimic of the 3′ end of aminoacyl-tRNA that prematurely terminates translation by substituting for the incoming aminoacyl-tRNA and acting as an acceptor either for the growing peptide chain donated by the P-site peptidyl-tRNA, or, during initiation, the fMet donated by P-site initiator tRNA (fMet-tRNA_i_^fMet^) (Figure 2A) (44). Using the puromycin reactivity of ^35^S-methionine-labeled 70S initiation complexes (70S IC) as a biochemical system, we followed the inhibition of PTC reactivity by antibiotics and PTC rescue by ABCF ARE factors. The 70S IC was formed from purified 70S ribosomes isolated from either *E. coli, S. aureus* or *E. faecalis*, a synthetic mRNA encoding the Met-Phe dipeptide (mRNA(MF)), and *E. coli* initiator tRNA that is aminoacylated with ^35^S-methionine and subsequently formylated (^35^S-fMet-tRNA_i_^fMet^) (42). Formation of the 70S ICs was catalyzed by *E. coli* initiation factors that were subsequently removed by ultracentrifugation through the sucrose cushion.

Since *E. coli* 70S ribosomes are intrinsically resistant to lincomycin (27,45), we opted for *S. aureus* 70S purified from the SH1000 model laboratory strain (46). Unlike *E. coli, S. aureus* ribosomes are highly sensitive to lincosamide antibiotics which are used to treat Staphylococcal infections in clinical practice (47), and several AREs have been identified as resistance factors in these bacteria. Already in moderate excess over ribosomes (0.7 μM antibiotic versus 0.5 μM 70S) both lincomycin (Figure 2B) and clindamycin (Figure 2C) completely abrogate the puromycin reactivity of *S. aureus* 70S IC. In the presence of saturating concentrations of lincomycin (0.7 μM and above) around 20% of ^35^S-methionine is released after 1000 s, reflecting slow dissociation of the drug followed by the puromycin reaction. Clindamycin near-completely inhibits puromycin reactivity even after a 1000-s incubation with puromycin, indicating even slower dissociation. Conversely, in the case of intrinsically lincomycin resistant *E. coli* 70S IC, puromycin releases ^35^S-methionine even in the presence of 5 or 15 μM lincomycin, resulting in a near-complete deacylation of the P-site ^35^S-fMet-tRNA_i_^fMet^ already by 200 seconds (Supplementary Figure S2A).

Since puromycin reacts with the P-site substrate relatively slowly, with a maximum rate of a 50 s^−1^ at pH 7.5 (48), it could potentially allow rebinding of the PTC blocker and, therefore, fail to reliably probe the dissociation kinetics of the antibiotic. Therefore, we tested the kinetic competitiveness of 1 mM puromycin against lincomycin added at the saturating concentration, 1 μM (Supplementary Figure S2B). When lincomycin is preincubated with ribosomes prior to the addition of puromycin, the puromycin reactivity is completely abrogated. However, when lincomycin is added simultaneously with puromycin, the PTC blocker abrogates a mere 5% of puromycin reactivity. This shows that when added in great excess over lincomycin, puromycin is a considerably faster binder and is, indeed, a reliable tool for probing the antibiotic dissociation from the ribosome.

Having established that the system is reliable, we tested the effect of 1 μM *S. haemolyticus* VgaA_LC_ on puromycin reactivity of *S. aureus* 70S IC inhibited by saturating concentrations of lincomycin (Figure 2D) and clindamycin (Figure 2E). In the absence of nucleotides, VgaA_LC_ has no effect on the puromycin kinetics. Conversely, in the presence of 1 mM ATP, VgaA_LC_ rescues the puromycin reactivity, although not completely. Bacterial antibiotic susceptibly tests demonstrated that *S. haemolyticus* VgaA_LC_ has specificity strongly shifted towards lincosamides and displays relatively poor activity against streptogramin A antibiotics (17). We have tested in our biochemical system a representative of the streptogramin A class, virgniamycin M1. The antibiotic is a poorer inhibitor of the puromycin reaction, requiring 10 μM concentration for complete inhibition at the 10 s time point (Supplementary Figure S3A). While addition of VgaA_LC_ has a clear ATP-dependent protective effect (Supplementary Figure S3C), the effect is not as pronounced as in the case of lincomycin. The pleuromutilin tiamulin is a moderately more efficient PTC inhibitor than virgniamycin M1, with inhibition efficiency saturating at 10 μM (Supplementary Figure S3B). Again, VgaA_LC_-mediated protection is strictly dependent on the presence of ATP (Supplementary Figure S3D).

### VgaA_LC_ is an NTPase that operates as a molecular machine, not as a molecular switch

**Figure 3.**
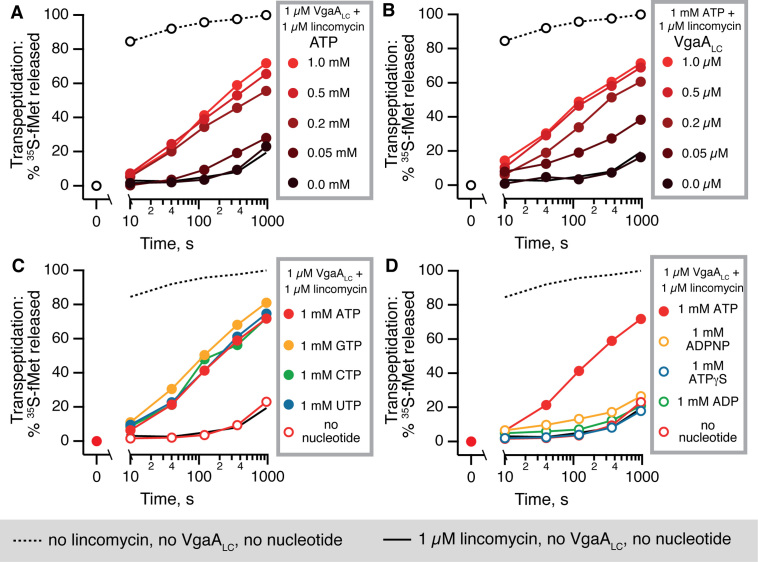
VgaA_LC_ activity requires mM-range concentrations of NTP nucleotides (ATP, GTP, CTP or UTP) and is not sustained either by the non-hydrolysable ATP analogue ADPNP, the slow-hydrolysable analogue ATPγS nor the product of ATP hydrolysis, ADP. Puromycin reactivity is progressively rescued by 1 μM VgaA_LC_ and increasing concentrations ATP (**A**) or by increasing concentrations VgaA_LC_ in the presence of constant 1 mM ATP (**B**). The resistance effect saturates at 0.5 mM ATP and 0.5 μM VgaA_LC,_ respectively. VgaA_LC_-mediated rescue of puromycin reactivity is supported by either ATP, GTP, CTP or UTP nucleotides added at 1 mM, suggesting that VgaA_LC_ is an NTPase, rather than a strict ATPase (**C**). Neither ADP, nor the non-hydrolysable ATP analogue ADPNP nor the slow-hydrolysable analogue ATPγS can support VgaA_LC_ activity, indicating that NTP hydrolysis, not just the NTP binding, are necessary (**D**).

In the following experiments we focused on ABCF-mediated protection from lincomycin. The addition of 1 μM VgaA_LC_ and 1 mM ATP rescues the puromycin reactivity inhibited by saturating concentration of the antibiotic (1 μM). However, the protection is not complete; the transpeptidation reaction is still significantly slower than in the absence of the antibiotic. One possible reason could be that the system is not saturated with either the nucleotide substrate, ATP, or VgaA_LC_. Therefore, we titrated ATP (Figure 3A) and VgaA_LC_ (Figure 3B) in the presence of 1 μM lincomycin. The protective effect reaches a plateau at 0.5 mM ATP and 0.5 μM VgaA_LC_, i.e. 1:1 ratio of ARE to 70 IC. Even in these conditions, puromycin reactivity is not completely restored. In the following puromycin reaction assays we used excess concentrations of VgaA_LC_ and nucleotides: 1 μM and 1 mM, respectively.

Next, we characterized the nucleoside triphosphate specificity of VgaA_LC_-mediated lincomycin resistance. The puromycin release kinetics is identical when ATP is substituted for GTP, CTP or UTP, suggesting that VgaA_LC_ is an NTPase (Figure 3C). In the absence of VgaA_LC_ none of the NTPs alone have an effect on the system (Supplementary Figure S4A). To characterize the NTP specificity of VgaA_LC_, we titrated GTP, the second most-abundant NTP species in bacteria after ATP (49) (Supplementary Figure S4B). Similarly to ATP, the full activity of VgaA_LC_ is achieved at 0.5 mM GTP, suggesting that in the cell the enzyme operates at saturating concentrations of both NTP species. Finally, to probe the role of NTP hydrolysis by VgaA_LC_, we compared ATP, its non-hydrolysable analogue ADPNP (adenosine 5′-(β-γ-imido) triphosphate, App(NH)p), a slow-hydrolysable analogue ATPγS (adenosine 5-*O*-(3-thio) triphosphate) and the product of ATP hydrolysis, ADP (Figure 3D). Neither ADPNP, nor ATPγS, nor ADP support VgaA_LC_ activity, suggesting driving the conformational switch by ATP analogues is not sufficient to rescue the PTC activity.

Since VgaA_LC_ can utilize GTP as a substrate, we tested if the alarmone nucleotide ppGpp could suppress the VgaA_LC_-mediated resistance acting as an orthosteric inhibitor the same way as it inhibits GTPases and nucleotide metabolism enzymes (50). During acute amino acid starvation, ppGpp becomes the dominant guanosine nucleotide, reaching sub-mM concentration (49). However, even when added at 0.5 mM, ppGpp does not have a pronounced inhibitory effect on VgaA_LC_ (Supplementary Figure S5).

### The hydrolytically inactive *S. haemolyticus* VgaA_LC_EQ_2_ and *E. faecalis* LsaAEQ_2_ mutants inhibit ribosomal peptidyl transferase in the presence of NTP

Use of non- or slowly-hydrolysable ATP analogues is one approach to generate an ATP-bound state of VgaA_LC_. An alternative is to mutate conserved catalytic glutamate residues following the Walker B motif in the two ABC cassettes and use the native ATP substrate. These glutamates form strong hydrogen bond interactions with the attacking water molecule, polarizing it as a nucleophile (51). Simultaneous mutation of the residues in both cassettes for glutamine (E105Q and E410Q; EQ_2_) results in a deficiency in ATP hydrolysis by ABC ATPases (52) and locks the enzyme in an ATP-bound active conformation (33,53).

**Figure 4.**
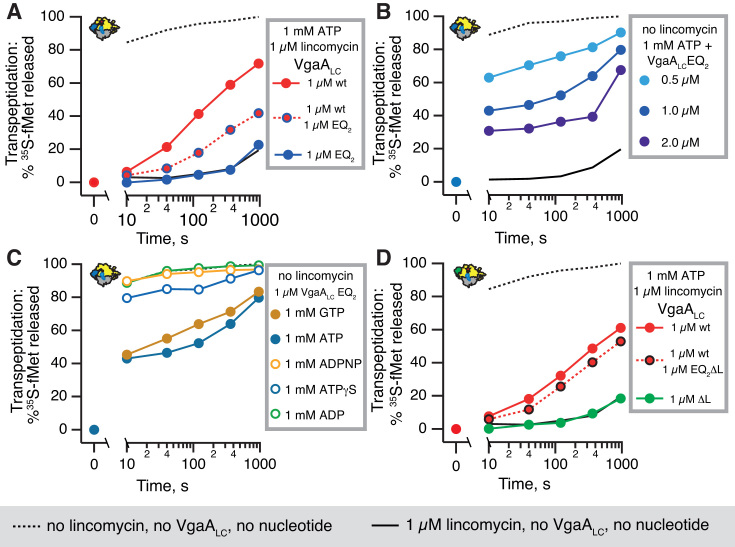
The linker region is essential for NTP-dependent inhibition of ribosomal peptidyl transferase activity by the NTP hydrolysis-incompetent VgaA_LC_EQ_2_ mutant. The NTPase-incompetent VgaA_LC_EQ_2_ mutant does not rescue the puromycin reactivity of *S. aureus* 70S IC(MF) inhibited by 1 μM lincomycin (dark blue filled circles), and compromises protection by the wild type protein (dark blue circles, red fill) (**A**). The VgaA_LC_EQ_2_ mutant inhibits IC(MF) puromycin reactivity in the absence of antibiotics in the presence of 1 mM ATP (B and C) or GTP (**C**), but not 1 mM ADP, ATPγS or ADPNP (**C**). The VgaA_LC_ΔL mutant in which inter-ABC linker region (amino acids K199-S226) is substituted for a GSG linker neither protects the puromycin reactivity from 1 μM lincomycin (filled green circles), and its EQ_2_ variant, VgaA_LC_EQ_2_ΔL, does not abolish the protective effect of the wild type protein (black circles with red fill) (**D**).

The hydrolytically deficient VgaA_LC_EQ_2_:ATP does not rescue inhibition of transpeptidation by lincomycin and compromises protection by the wild type VgaA_LC_ (Figure 4A). A possible explanation is that NTPase-inactive VgaA_LC_EQ_2_ competes with the wild type for binding to the ribosome, thus preventing the wild type from carrying out its protective function. A more non-trivial scenario is that in addition to competing with the wild type, VgaA_LC_EQ_2_ actively inhibits transpeptidation. Therefore, we tested the effects of VgaA_LC_EQ_2_ on transpeptidation in the absence of lincomycin. The addition of increasing concentrations of VgaA_LC_EQ_2_ in the presence of ATP progressively inhibits the puromycin reaction, reducing it by ≈70% in the presence of 2 μM protein (Figure 4B). Just like the protective effect of the wild type protein, PTC inhibition by VgaA_LC_EQ_2_ requires NTP (ATP and GTP were tested), and is not supported by ADP, ADPNP or ATPγS (Figure 4C).

**Figure 5.**
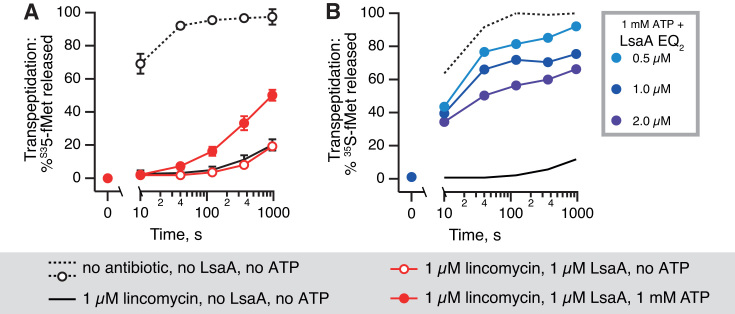
Wild type *E. faecalis* LsaA ATP-dependently protects *E. faecalis* 70S initiation complexes from lincomycin antibiotics, and the NTP hydrolysis-incompetent LsaAEQ_2_ mutant inhibits the peptidyl transferase activity. In the presence of 1 mM ATP (filled red circles) 1 μM *E. faecalis* LsaA rescues the puromycin reactivity inhibited by 1 μM lincomycin; in the absence of ATP LsaA has no effect (empty red circles) (**A**). Increasing concentrations of LsaAEQ_2_ progressively inhibit the transpeptidase activity of *E. faecalis* 70S initiation complexes (**B**).

To test whether PTC inhibition is a general feature of EQ_2_ versions of ABCF ARE factors, we characterized *E. faecalis* LsaA. Although the efficiency is lower than *S. haemolyticus* VgaA_LC_, wild type LsaA protects *E. faecalis* 70S IC from inhibition by lincomycin (Figure 5*A*). Similarly to VgaA_LC_, The EQ_2_ mutant inhibits the transpeptidation activity of *E. faecalis* 70S IC in the presence of ATP (Figure 5*B*). Note that unlike *E. faecalis* LsaA or *S. haemolyticus* VgaA_LC_, the EQ_2_ mutant of *E. coli* housekeeping ABCF EttA does not inhibit the PTC activity directly as manifested by unperturbed formation of the first peptide bond (32).

### Deletion of the linker region renders wild type VgaA_LC_ inactive in antibiotic protection and the EQ_2_ mutant inactive in PTC inhibition

The likely structural candidate responsible for antibiotic displacement by wild type VgaA_LC_ and PTC inhibition by the EQ_2_ mutant is the extended inter-ABC linker region (21) (Figure 1). This hypothesis, however, has never been tested experimentally. In the absence of structural information on VgaA_LC_ it is impossible to design a ‘clean’ deletion of the linker region. We designed a deletion that reduces the length of the linker to that seen for EttA. In this truncated VgaA_LC_ΔL mutant, amino acids K199-S226 are substituted for a ‘stump’ formed by a flexible GSG linker. Additionally, the ΔL deletion was combined with the EQ_2_ double point mutation (E105Q and E410Q) to inactivate hydrolytic activity. Deletion of the linker region has a profound effect on the functionality of VgaA_LC_: neither does VgaA_LC_ΔL protect from lincomycin nor does VgaA_LC_EQ_2_ΔL compromise the protective effect of the wild type VgaA_LC_ (Figure 4D). This suggests that the truncated protein can no longer reach and interact with the PTC region (Supplementary Figure S1). However, it is also possible that the deletion alters the overall structural integrity of VgaA_LC_ thus inhibiting the protein's activity indirectly, e.g. by abrogating its binging to the ribosome or/and ATPase activity. Detailed structural information is necessary to guide precise structure-function characterization of ARE ABCF factors.

## DISCUSSION

**Figure 6.**
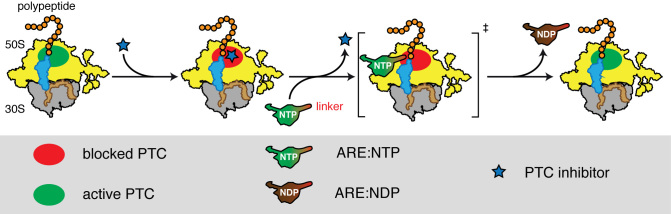
Model of ARE-mediated ribosome protection from antibiotics targeting the PTC. LS_A_ antibiotics directly bind to the PTC and inhibit its catalytic activity. ARE factors dislodge the antibiotics in an NTP-dependent manner. Association of the ARE with the ribosome causes transient arrest of transpeptidation, conceivably via a direct contact of the linker region with the PTC.

We suggest a model of ARE-mediated ribosome protection from PTC inhibition by LS_A_ antibiotics (Figure 6). Powered by NTP-hydrolysis, ARE factors dislodge LS_A_ antibiotics from the ribosome. In the cell, AREs can use any NTP species, the most abundant being GTP and ATP. The association of the NTP-bound ARE with the ribosome causes transient arrest of transpeptidation, conceivably via a direct contact of the linker region with the PTC.

The relaxed nucleotide substrate specificity seen with VgaA_LC_ is not unusual for ABC enzymes, e.g. *E. coli* transporter CvaB uses both GTP and ATP (54), and ribosome-associated ABCF EttA was mentioned by Böel and colleagues to interact with both ATP and GTP (no data shown in the original publication) (32). To achieve its maximum activity, VgaA_LC_ requires ≥500 μM of the NTP substrate. While this is a relatively high concentration, it follows the general trend of enzyme affinity to substrate correlating with the concentration of substrate (55): there is no evolutionary pressure selecting for high affinity to abundant substrates. Since two NTP species—GTP and ATP—are present in bacterial cell in the mM concentration range (49), it is not surprising that in the test tube VgaA_LC_ requires high NTP concentration.

Our biochemical analyses raise several pertinent questions that can be addressed by structural and microbiological approaches. First, high resolution structural information is necessary to unequivocally establish the role of the ‘linker’ region in ARE’s molecular mechanism. Second, the low efficiency of protection by VgaA_LC_ in the reconstituted system indicates that the biochemical experiments do not reveal the full story. One possible explanation could be that in the test tube, the dislodged antibiotic rapidly rebinds to the ribosome, thus compromising VgaA_LC_-mediated protection. However, this is unlikely, since in direct competition experiments the puromycin test reaction is sufficiently fast to kinetically outcompete the slowly binding lincomycin (Supplementary Figure S2B). A second explanation is that the reconstituted system is missing one or more components or co-factors crucial for the full activity of VgaA_LC_. It is tempting to speculate that in order to achieve efficient removal of the dislodged antibiotic, the activity of the ABCF ARE is coordinated with a direct interaction of the ARE with an efflux pump. Experiments with *Streptococcus pneumoniae* macrolide resistance ARE MsrD indicate a possible candidate. The *msrD* gene is co-transcribed with another ORF encoding MefE (macrolide efflux E) efflux pump, a member of major facilitator superfamily (56). While acting alone in the absence of MefE, the MsrD ABCF confers only low-level macrolide resistance (57), but the two proteins synergize when acting together (58). Although they are Streptococcal resistance factors, MefE and MsrD confer macrolide resistance in *E. coli*, suggesting that the proteins are functional when expressed heterologously (59). While GFP-labelled MsrD localized in the *E. coli* cytoplasm, GFP-labelled MefE is localized at cell poles—but only when co-expressed with wild type MsrD. This indicates a physical interaction between the two proteins. However, just how general this kind of interaction might be among ABCF AREs is unknown. Genetic, microbiological and molecular biology experiments with Staphylococcal VgaA are the next step for identification of its potential interactive partners, paving the way for more refined biochemical and structural studies.

## Supplementary Material

Supplementary Data
