# Supplementary material for: Antibiotic resistance ABCF proteins reset the peptidyl transferase centre of the ribosome to counter translational arrest
Source: Nucleic Acids Res. 2018 Feb 5;46(7):3753–63. doi: 10.1093/nar/gky050 (PMC5909423; doi:10.1093/nar/gky050)
Supplement: Supplementary Data [file gky050_supp.docx]

**Supplementary Information**

**Antibiotic resistance ABCF proteins reset the peptidyl transferase center of the ribosome to counter translational arrest**

Victoriia Murina^1,2^, Marje Kasari^1^, Vasili Hauryliuk^1,2,3,*^, Gemma C. Atkinson^1,*^

^1^Department of Molecular Biology, Umeå University, 901 87 Umeå, Sweden

^2^Laboratory for Molecular Infection Medicine Sweden (MIMS), Umeå University, 901 87 Umeå, Sweden

^3^University of Tartu, Institute of Technology, 50411 Tartu, Estonia

^*^ to whom correspondence should be addressed:

Vasili Hauryliuk, Email: vasili.hauryliuk@umu.se, Tel. +46 706090493

Gemma C. Atkinson, Email: gemma.atkinson@umu.se, Tel. +46 706070315

**Supplementary methods**

*Preparation of 10X Polymix buffer base (also reference (1))*

For preparation of the putrescine solution, 100 g of putrescine (1,4-diaminobutane) was dissolved in 600 mL of ddH_2_O at 90°C, and the pH adjusted with acetic acid to 8.0 (approximately 100 mL of 100% acetic acid). After cooling to room temperature, the pH was adjusted further to 7.6 and the volume was adjusted to the final of 2 L by addition of 1.134 L of ddH_2_O. One 100 mL cup of activated charcoal was added and the slurry was stirred under the hood for 30 minutes. The slurry was filtered through, first, Whatman paper and then through a 0.45 µm BA85 membrane. The final solution was stored at 4°C in a bottle wrapped in foil since putrescine is photosensitive.

The preparation of 2 L of 10X Polymix buffer base used 141.66 g KCl, 5.35 g NH_4_Cl, 21.44 g Mg(OAc)_2_•4H_2_O, 1.47 g CaCl_2_•2H_2_O, 5.092 g spermidine, and 160 ml of putrescine solution (described above). The salts were dissolved in ddH_2_O (~1500 ml), then the putrescine solution was added and mixed well. Spermidine was dissolved in a small volume of ddH_2_O and added to the mixture. The pH was adjusted to 7.5 with concentrated acetic acid or 5M KOH, and after that the volume was adjusted by adding ddH_2_O to 2L. The buffer was filtered through 0.2 μm nitrocellulose filter (2-3 filters are needed). The resulting 10X Polymix buffer base was aliquoted and stored at -20°C.

The final working HEPES:Polymix buffer was made using the 10X Polymix buffer base, 1M DDT and 1 M HEPES:KOH pH 7.5 and contains 20 mM HEPES:KOH pH 7.5, 2 mM DTT, 5 mM MgOAc_2_, 95 mM KCl, 5 mM NH_4_Cl, 0.5 mM CaCl_2_, 8 mM putrescine, 1 mM spermidine. Note that for preparation of a phosphate-based Polymix buffer, the 10X Polymix buffer base should be first diluted with ddH_2_O before adding 1M potassium phosphate buffer (20X stock): mixing the 20X phosphate stock directly with 10X Polymix buffer base results in precipitation.

*Preparation of N10-formyltetrahydrofolate (10-THF) solution*

25 mg of folinic acid (Sigma) was dissolved in 2 mL of 50 mM β-mercaptoethanol, 220 μL of 1 M HCl was added and the reaction mix was incubated in darkness at room temperature for 3 hours. The solution was aliquoted (150 μL) and kept at -80°C for long-term storage. Prior to use, the pH of the solution was adjusted to pH 7.5 with approximately 1/10 volume of 1 M KOH and 1/50 volume 1 M HEPES:KOH (the latter is used for buffering capacity), and the pH-adjusted solution was stored on ice prior to use.

*Synthesis of benzoylated DEAE Sepharose Fast Flow used for purification of fMet-tRNA_i_^Met^*

This matrix combines ion exchange (DEAE) and hydrophobic (benzoyl) properties to specifically separate aminoacylated and formylated tRNA species. The synthesis requires the following reagents: 1 L DEAE of Sepharose Fast Flow (GE Healthcare), 14 L EtOH 95%, 5.5 L EtOH 99.5%, 1 L dimethylformamide (DMF), 1 L pyridine, 2 L toluene, 0.4 L benzoylchloride, 350 g NaCl and 5 ml 12 M HCl. The necessary equipment includes: 3 L sintered glass frit funnel, 5 L E-flask, 2 L glass beaker, rubber gasket, rubber stopper, glass rod, 3 L three necked round bottomed flask, thermostated water bath, variable-speed stirrer equipped with stainless steel and a teflon stirring rod, reflux cooler and a spoon.

Prior to carrying out the substitution with benzoyl chloride, the DEAE resin was washed with an EtOH gradient in order to remove water from the gel. 1 L DEAE Sepharose Fast Flow was resuspended and gently poured onto a sintered glass frit funnel, placed on a rubber gasket on top of an E-flask. The gel was washed in succession with 2 L solutions of increasing concentration of EtOH: 20%, 40%, 60%, 80%, 95% and 99.5%. The solutions were poured on a spoon held on top of the surface of the sedimented gel to prevent penetration of its surface. The gel surface was kept wet all the time. The next EtOH solution was poured onto the gel when there was an approximately 5 mm depth layer left of the preceding liquid on top of the gel. The gel was then resuspended in 0.5 L EtOH 99.5% and gently transferred to a 2 L glass beaker that was covered with aluminium foil and kept overnight.

The substitution reaction itself is performed in a well-ventilated fume cupboard. The water-free gel suspension in 99.5% EtOH was again poured into the sintered glass funnel. After draining, the gel was washed with 1 L dimethylformamide and drained. Finally, the gel was washed with 1.5 L of a pyridine-toluene mixture (1:2). After resuspension in 0.2 L of the pyridine-toluene mixture the gel was transferred to a three necked 3 L round bottom reaction flask equipped with a variable speed stirrer (stainless steel shaft and teflon blade) and a reflux cooler and placed in a thermostated water bath. The stirrer was set to 500 rpm during the whole course of the reaction. 200 mL pyridine-toluene mixture was added followed by 100 mL of benzoyl chloride. When the initial reaction had ceased, another 200 mL pyridine-toluene mixture was added, followed by 300 mL benzoyl chloride, added in small portions. The temperature of the water bath was slowly (in 30 min) raised to 70°C and was kept at this temperature for 5 hours when the reaction mixture was cooled down to room temperature using cold tap water added to the water bath. The stirring was then stopped and the gel suspension allowed to settle for 30 min. The supernatant liquid layer was decanted and the stirring started. 1 L 99.5% EtOH was added in small portions. The temperature raised to 54°C but was cooled to below 30°C using cold tap water and stirring was continued for another 30 min. The gel was then poured into the sintered glass funnel and washed with a decreasing gradient of EtOH. 1.5 L 99.5% EtOH was required to reduce the colour of the gel to its original white, followed by 6 x 600 mL 95 % EtOH, 1 L 80% EtOH, 1 L 60 % EtOH, 1 L 40% EtOH + 1 M NaCl + 50 mM HCl, 5 L 40 % EtOH + 1 M NaCl, 7x1 L distilled water. After having added the 7th L distilled water a check for remaining chloride ions with AgNO_3_ was made and the gel was poured in a bottle for storage at 4°C before packing into a column.

*Preparation of* E. coli *fMet-tRNA_i_^Met^*

The starting material, deacylated *E. coli* initiator tRNA_i_^Met^ was purchased from Chemical Block. The 400-1000 μL reaction mixture containing 100 μM tRNA_i_^Met^, 1 mM N10-formyltetrahydrofolate (10-THF), 2 μM methionyl-tRNA synthetase (Met-RS), 2 μM methionyl-tRNA formyltransferase (FMT), 2.5 mM ATP, 3 mM β-mercaptoethanol and 200 μM of ^35^S-methionine (specific activity 5000 cpm/pmol) was incubated at 37°C for 15 minutes, and then the reaction was stopped by addition of 10 mL of cold **loading buffer** containing 20 mM NaOAc pH 5.1, 1 mM EDTA, 5 mM β-mercaptoethanol. The solution was loaded onto benzoylated DEAE Sepharose Fast Flow column (100 mL) equilibrated with the **loading buffer**. ^35^S-formyl-methionyl-tRNA_i_^Met^ (^35^S-fMet-tRNA^Met^) was eluted with 450 mL NaCl gradient in the same buffer, from 0.02 to 1.0 M. The peak that elutes at conductivity of 77 mS/cm was collected, supplemented with 2.5 volumes of 95% ethanol and 0.1 volume 3 M NaOAc pH 5.1, and precipitated at -20°C overnight.

^35^S-labelled fMet-tRNA^Met^ was pelleted by centrifugation at 20,000 rpm during 40 minutes 4°C (45 Ti, Beckman) and the pellet was resuspended in 3 mL of 5 mM NaOAc pH 5.1. Next, 3 volumes of 95% ethanol and 0.1 volume of 3 M NaOAc pH 5.1 were added to the solution and tRNA was precipitated again at -20°C overnight or 30 minutes at -80°C. ^35^S-fMet-tRNA^Met^ was pelleted by centrifugation at 20,000 rcf for 20 minutes at 4°C, washed with ice-cold 75% EtOH to remove the precipitated salt, resuspended in 100 μL of 5 mM KOAc pH 5.1, and stored at -80°C. The concentration of tRNA was measured by absorbance at OD_260_, taking into account that 1.0 OD_260_ corresponds to 1 μM tRNA_i_^Met^.

*Protein cloning, expression and purification*

Expression constructs for *E. coli* methionyl-tRNA synthetase (MetRS), methionyl-tRNA formyltransferase (FMT) and initiation factors IF1, IF2 and IF3 are described in (2).

**Ubiquitin-like-specific protease 1, Ulp1**: The expression plasmid for Ulp1, pCA535 (3) was kindly provided by Dr. Claes Andréasson (Stockholm University). For protein expression the plasmids were transformed into *E. coli* BL21 DE3 Rosetta (Novagen). Transformants were selected on LB plates supplemented with 50 mg/ml kanamycin and 25 mg/ml chloramphenicol. An overnight culture of freshly transformed *E. coli* BL21 DE3 Rosetta was diluted to OD_600_ of 0.06 in 2xYT media supplemented with 50 mg/ml kanamycin and 25 mg/ml chloramphenicol, cells were grown at 37°C until OD_600_ of 0.8, transferred to 21°C and protein expression was induced with 0.5 mM IPTG (final concentration) for 16 hours at 21°C. Cells were collected by centrifugation, resuspended in **loading buffer (buffer A)** (150 mM KCl, 40 mM HEPES pH 7.4, 2 mM β-mercaptoethanol) supplemented with 0 1 U/ml DNAse 1. Cells were lysed by a Stansted Fluid Power SPCH ultra high-pressure cell disrupter/homogenizer and cell debris was removed by centrifugation (35,000 rpm for 30 minutes). The supernatant was loaded onto 1 mL HisTRAP HP (GE Healthcare) column pre-equilibrated with **buffer A** and eluted with a gradient of **loading buffer supplemented with 0.5 M imidazole (buffer B)** (150 mM KCl, 40 mM HEPES pH 7.4, 500 mM imidazole, 2 mM β-mercaptoethanol; 0 to 100% gradient, 50 column volumes). The fractions containing Ulp1 protein (eluting in 25-35% **buffer B**) were combined, concentrated and exchanged into **buffer A** on 3 MWCO centricons (Amicon). The resulting protein solution was supplemented with 50% glycerol (final concentration) aliquoted and stored at -80°C. The purity of the protein was confirmed by SDS-PAGE and OD_280_/OD_260_ ratio of 1.9.

***S. haemolyticus* VgaA_LC_**: All cloning was performed by the Protein Expertise Platform at Umeå University. The *S. haemolyticus* VgaA_LC_ ORF encoded on the pRB374 VgaA_LC_ plasmid (4) was kindly provided by Dr. Gabriela Balíková Novotná. The gene was sub-cloned to the pET24d *E. coli* expression vector with a C-terminal 6His tag preceded by a single glycine linker. Point mutations E105Q and E410Q were introduced to the plasmid to make an ATPase deficient mutant, the so called EQ_2_ mutant. The linker deletion mutant (ΔL) was made based on sequence alignment between EttA and VgaA_LC_. Amino acids K199-A226 were replaced with the sequence GSG. EQ_2_ mutations at equivalent sites to those made for wild type VgaA_LC_, E105Q and E385Q, were introduced to the pET24d VgaA_LC_ ΔL plasmid.

*E. coli* BL21 DE3 Rosetta pET24d cells, having been freshly transformed with the resultant pET24d VgaA_LC_ (wt, EQ2 or Δlinker) expression construct were inoculated overnight in LB media supplemented with 50 mg/ml kanamycin and 25 mg/ml chloramphenicol. Next morning the overnight culture was diluted to OD_600_ of 0.06 in the same media, the culture was grown at 37°C until an OD_600_ of 0.6-0.7 and induced with 1 mM IPTG (final concentration) and grown for an additional two hours at 30°C. The cells were harvested by centrifugation, resuspended in **lysis buffer** **(buffer A)** (1 M NaCl, 100 mM Tris:HCl pH 7.5, 10 mM MgCl_2_, 10% glycerol, 5 mM imidazole and 2 mM β-mercaptoethanol) supplemented with 0.1 mM PMSF protease inhibitor, 35 µg/ml lysozyme and 1 u/ml DNase I. Cells were lysed by Stansted Fluid Power SPCH ultra high-pressure cell disrupter/homogenizer, the cell debris removed by centrifugation (35,000 rpm, 40 minutes) and the supernatant was loaded onto 1 mL HisTRAP HP (GE Healthcare) column equilibrated in **lysis buffer** **(buffer A)**. The column was washed with **high salt buffer (buffer B)**, to remove RNA contamination (2 M NaCl, 100 mM Tris:HCl pH 7.5, 25 mM imidazole, 10 mM MgCl_2_, 2 mM β-mercaptoethanol) and the protein was eluted with the **loading** **buffer** **supplemented with 0.5 M imidazole (buffer C)** (0.7 M KCl, 0.5 M imidazole, 50 mM Tris:HCl pH 7.5, 10 mM MgCl_2_, 2 mM β-mercaptoethanol).

Although after HisTRAP chromatography VgaA_LC_ preparations (wt and mutants) were pure as judged by an SDS PAGE protein gel, the protein was contaminated with RNA (OD_280_/OD_260_ of around 1.0). Therefore, the protein was subjected to anion exchange chromatography (HiPrep Q XL 16/10 20 ml, GE Healthcare). The protein was buffer exchanged using 10 MWCO centricons (Amicon) into **low salt buffer** (100 mM NaCl, 50 mM Tris:HCl pH 7.5, 5 mM MgCl_2_, 2 mM β-mercaptoethanol), and loaded onto the column equilibrated in the same buffer. At this step the protein passes though the column, while RNA is efficiently captured. The flow-through fraction is concentrated on 10 MWCO centricons (Amicon) and the buffer is exchanged for **storage buffer** (350 mM KCl, 25 mM HEPES pH 7.5, mM MgCl_2_, 2 mM β-mercaptoethanol, 50% glycerol). The purity of the protein was confirmed by SDS-page and an OD_280_/OD_260_ ratio of around 1.8. The protein was stored at -20°C.

***E. faecalis* LsaA**: All cloning was performed by Protein Expertise Platform at Umeå University. *E. faecalis* LsaA ORF was PCR amplified from pTEX5333 plasmid (5) kindly provided by Dr. Barbara E. Murray (University of Texas, Health Science Center) using CCAGTGGGTCTCAGGTGGTTCGAAAATTGAACTAAAACAAC (forward) and ccttaaggatccttaTGATTTCAAGACAATTTTTTTATCC (reverse) primers and cloned into pCA528 for 6His-SUMO-tagging (3) kindly provided by Dr. Claes Andréasson (Stockholm University) using BsaI and BamH restriction enzymes resulting in pET24d_6His-SUMOLsaA. Point mutations E142Q and E452Q were introduced to p6His-SUMOLsaA using point mutagenesis resulting in p6His-SUMO-LsaAEQ_2_.

For protein expression the plasmids were transformed into *E. coli* BL21 DE3 Rosetta (Novagen). Transformants were selected on LB plates supplemented with 50 mg/ml kanamycin and 25 mg/ml chloramphenicol. An overnight culture of freshly transformed *E. coli* BL21 DE3 Rosetta was diluted to OD_600_ of 0.06 in LB media supplemented with 50 mg/ml kanamycin and 25 mg/ml chloramphenicol, cells were grown at 37°C until OD_600_ of 0.6-0.7 and protein expression was induced with 1 mM IPTG (final concentration) for two hours at 30°C. Cells were collected by centrifugation, resuspended in **lysis buffer** (0.7 M KCl, 100 mM HEPES pH 7.5, 10 mM MgCl_2_, 10 mM imidazole, 10% glycerol, 2 mM β-mercaptoethanol, 0.1% Tween 20) supplemented with 0.1 mM PMSF protease inhibitor, 35 µg/ml lysozyme and 1 U/ml DNAse 1. Cells were lysed by a Stansted Fluid Power SPCH ultra high-pressure cell disrupter/homogenizer and cell debris was removed by centrifugation (35,000 rpm for 40 minutes). The supernatant was loaded onto 1 mL HisTRAP HP (GE Healthcare) column pre-equilibrated with **loading buffer (buffer A)** (0.7 M KCl, 50 mM HEPES pH 7.5, 5 mM MgCl_2_, 10 mM imidazole, 2 mM β-mercaptoethanol). After sample loading column was washed with 20 column volumes of **high salt buffer (buffer B)** (2 M KCl, 50 mM HEPES pH 7.5, 20 mM imidazole, 5 mM MgCl_2_, 2 mM β-mercaptoethanol) and eluted with a gradient of **loading buffer supplemented with 0.5 M imidazole (buffer C)** (0.7 M KCl, 50 mM HEPES pH 7.5, 5 mM MgCl_2_, 500 mM imidazole, 2 mM β-mercaptoethanol; 0 to 50% gradient, 50 column volumes).

The fractions containing 6His-SUMO-tagged LsaA (wt or EQ_2_) protein (eluting in 10-25% **buffer C**) were combined and diluted to a final concentration of KCl of 500 mM (maximum salt concentration compatible with SUMO cleavage by Ubiquitin-like-specific protease 1, Ulp1) in **dilution buffer** (50 mM HEPES pH 7.5, 5 mM MgCl_2_, 10% glycerol, 2 mM β-mercaptoethanol) and 35 µg of Ulp1 per 1 mg of 6HisSUMO-tagged LsaA (wt or EQ_2_ mutant) was added. The 6HisSUMO tag was cut off during buffer exchange to **loading buffer (buffer A2)** (500 mM KCl, 50 mM HEPES pH 7.5, 5 mM MgCl_2_, 10% glycerol, 2 mM β-mercaptoethanol) using 3 MWCO centricons (Amicon) during 1 hour at 19°C. The mixture was loaded onto 1 mL HisTRAP HP column (GE Healthcare) pre-equilibrated with **buffer A** (0.7 M KCl, 50 mM HEPES pH 7.5, 5 mM MgCl_2_, 10 mM imidazole, 2 mM β-mercaptoethanol) to remove the uncut 6HisSUMO-tagged LsaA as well as Ulp1 protease. The flow-through containing either tagless LsaA or LsaAEQ_2_ was collected. To remove the RNA contamination from the sample it was diluted to 300 mM KCl (final concentration) in **dilution buffer** and loaded to anion exchange column (HiPrep Q XL 16/10 20 ml, GE Healthcare) pre-equilibrated with **mid-salt buffer** (300 mM KCl, 50 mM HEPES pH 7.5, 5 mM MgCl_2_, 2 mM β-mercaptoethanol). Neither LsaA nor LsaAEQ_2_ bind to the column, while RNA is efficiently captured. The flow-through from column was collected, concentrated and exchanged into **storage buffer** (350 mM KCl, 25 mM HEPES pH 7.5, 10 mM MgCl_2_, 2 mM β-mercaptoethanol and 50% glycerol) on 10 MWCO centricons (Amicon). The resulting proteins were aliquoted and stored at -20°C. The purity of the protein was confirmed by SDS-page and OD_280_/OD_260_ ratio of 1.6-1.8.

***E. coli MetRS***: *E. coli* BL21 (DE3), transformed with the expression construct for C-terminally His-tagged MetRS was grown in LB medium supplemented with 100 mg/ml ampicillin at 37°C until OD_600_ reached 0.5. Protein expression was induced with 1 mM IPTG (final concentration) and cells were grown for an additional 8 hours at 30°C. Cells were collected by centrifugation and frozen with liquid nitrogen.

For purification, 30 g of overexpressing cells were resuspended in **lysis buffer (buffer A)** (150 mM NaCl, 50 mM Tris:HCl pH 7.5, 5 mM MgCl_2_, 20 mM imidazole and 1 mM β-mercaptoethanol), lysed by Stansted Fluid Power SPCH ultra high-pressure cell disrupter/homogenizer, the cell debris removed by centrifugation (35,000 rpm, 40 minutes) and the supernatant was loaded onto a 5 mL HisTRAP HP column (GE Healthcare) equilibrated in **buffer A.** To remove the RNA contamination, the column was washed with **high salt buffer (buffer B)** (1 M NaCl, 50 mM Tris:HCl pH 7.5, 20 mM imidazole, 5 mM MgCl_2_, 1 mM β-mercaptoethanol), and the protein was eluted with a 40 mL gradient of **lysis** **buffer supplemented with 0.5 M imidazole (buffer C)** (150 mM NaCl, 0.5 M imidazole, 50 mM Tris:HCl pH 7.5, 5 mM MgCl_2_, 1 mM β-mercaptoethanol). Fractions containing pure MetRS were pooled, and the buffer was exchanged for **storage buffer** (50 mM KCl, 50 mM HEPES pH 7.5, 15 mM MgCl_2_, 1 mM β-mercaptoethanol, 10% glycerol) on HiPrep 26/10 desalting column (GE Healthcare). The protein was concentrated on 50 MWCO centricons (Amicon). The purity of the protein was confirmed by SDS-PAGE. The protein was aliquoted and stored at -80°C.

***E. coli FMT***: *E. coli* BL21 (DE3), transformed with the expression construct for C-terminally His-tagged FMT was grown in LB supplemented with 100 mg/ml ampicillin at 37°C until OD_600_ reached 0.5, protein expression was induced with 1 mM IPTG (final concentration) and cells were grown for an additional 8 hours at 30°C. Cells were collected by centrifugation and frozen with liquid nitrogen.

For purification, 10 g of overexpressing of cells were resuspended in **lysis buffer** **(buffer A)** (150 mM NaCl, 20 mM Tris:HCl pH 7.5, 5 mM MgCl_2_, 20 mM imidazole and 1 mM β-mercaptoethanol), lysed by Stansted Fluid Power SPCH ultra high-pressure cell disrupter/homogenizer, the cell debris removed by centrifugation (40,000 rpm, 60 minutes) and the supernatant was loaded onto 2 mL HisTRAP HP (GE Healthcare) column equilibrated in **buffer A**. The column was washed with **high salt buffer (buffer B)**, to remove RNA contamination (1 M NaCl, 50 mM Tris:HCl pH 7.5, 20 mM imidazole, 5 mM MgCl_2_, 1 mM β-mercaptoethanol) and the protein was eluted with 20 mL gradient **lysis** **buffer supplemented with 0.5 M imidazole (buffer C)** (150 mM NaCl, 0.5 M imidazole, 50 mM Tris:HCl pH 7.5, 5 mM MgCl_2_, 1 mM β-mercaptoethanol). Fractions containing pure FMT were pooled and the buffer was exchanged for **storage buffer** (50 mM KCl, 50 mM HEPES pH 7.5, 15 mM MgCl_2_, 1 mM β-mercaptoethanol, 10% glycerol) on a desalting column HiPrep 26/10 (GE Healthcare). The protein was concentrated on 10 MWCO centricons (Amicon) and the purity confirmed by SDS-PAGE. The protein was aliquoted and stored at -80°C.

***E. coli IF1***: For protein expression, the pET24a plasmid encoding 6His-tagged IF1 was transformed into *E. coli* BL21 (DE3) (Novagen). Transformants were selected on LB plates supplemented with 50 mg/ml kanamycin. An overnight culture of freshly transformed *E. coli* BL21 (DE3) was diluted to OD_600_ of 0.06 in LB media supplemented with 50 mg/ml kanamycin, cells were grown at 37°C until OD_600_ of 0.6-0.7, protein expression was induced with 1 mM IPTG (final concentration) and cells were grown for an additional 7 hours at 30°C. Cells were collected by centrifugation.

For purification, 2 g of cells were resuspended in **lysis buffer (buffer A)** (1 M NaCl, 100 mM Tris:HCl pH 7.5, 5 mM MgCl_2_, 20 mM imidazole, 1 mM β-mercaptoethanol) supplemented with 0.1 mM PMSF protease inhibitor, 35 µg/ml lysozyme and 1 U/ml DNAse 1. Cells were lysed by a Stansted Fluid Power SPCH ultra high-pressure cell disrupter/homogenizer and cell debris was removed by centrifugation (40,000 rpm for 40 minutes). The supernatant was loaded onto a 1 mL HisTRAP HP (GE Healthcare) column pre-equilibrated with **buffer A**. The column was washed with **high salt buffer (buffer B)** (2 M NaCl, 50 mM Tris:HCl pH 7.5, 20 mM imidazole, 5 mM MgCl_2_, 2 mM β-mercaptoethanol), and then the protein was eluted with a gradient of **0.5 M imidazole buffer (buffer C)** (150 mM NaCl, 20 mM Tris:HCl pH 7.5, 5 mM MgCl_2_, 0.5 M imidazole, 1 mM β-mercaptoethanol). Fractions containing pure protein were pooled, diluted in **low salt buffer** (**buffer D**) (100 mM NH_4_Cl, 20 mM Tris:HCl pH 7.5, 5 mM MgCl_2,_  1 mM β-mercaptoethanol) and loaded onto anion exchange column HiPrep Q XL 16/10 (GE Healthcare) pre-equilibrated in **buffer D with 1 M NH_4_Cl** (final concentration) to remove RNA contamination. In these conditions the protein was not bound to the column and remained the in flow-through, while RNA was efficiently captured. The protein was concentrated on 10 MWCO centricons (Amicon) and the purity was confirmed by SDS-PAGE. The protein was aliquoted and stored at -80°C.

***E. coli IF2***: For protein expression, the pET24a plasmid encoding 6His-tagged IF2 was transformed into *E. coli* BL21 (DE3) (Novagen). Transformants were selected on LB-agar with 50 μg/mL kanamycin as a selectivity marker. Several colonies were inoculated in 50 mL of LB supplemented with 50 μg/mL kanamycin for overnight culturing at 37°C. The next day the overnight culture was diluted to OD_600_ 0.05 in LB supplemented with 50 μg/mL kanamycin and grown at 37°C to OD_600_ 0.6. Protein expression was induced with 1 mM IPTG (final concentration) and grown for an additional 2 hours at 30°C. Cells were collected by centrifugation and frozen with liquid nitrogen.

For purification, cells were resuspended in **loading buffer** (150 mM NaCl, 20 mM Imidazole, 50 mM Tris:HCl pH 7.5, 5 mM MgCl_2_, 1 mM β-mercaptoethanol) supplemented with 0.1 mM PMSF protease inhibitor and 1 U/ml DNAse 1. Cells were lysed by a Stansted Fluid Power SPCH ultra high-pressure cell disrupter/homogenizer and cell debris was removed by centrifugation (40,000 rpm for 40 minutes). The supernatant was loaded onto a 1 mL HisTRAP HP (GE Healthcare) column pre-equilibrated with **loading buffer**. After sample loading the column was washed with 12 column volumes of **loading buffer supplemented with additional 0.55 M NaCl (buffer B)** (0.7 M NaCl, 50 mM Tris:HCl pH 7.5, 20 mM imidazole, 5 mM MgCl_2_, 2 mM β-mercaptoethanol) and the protein was eluted with a gradient of **buffer supplemented with 0.5 M imidazole (buffer C)** (150 mM NaCl, 20 mM Tris:HCl pH 7.5, 5 mM MgCl_2_, 0.5 M imidazole, 1 mM β-mercaptoethanol). Eluted protein was collected, diluted 10 times in **low salt buffer** (**buffer D**) (30 mM NaCl, 30mM NH_4_Cl, 40 mM Tris:HCl pH 7.5, 5 mM MgCl_2,_  1 mM β-mercaptoethanol) and loaded onto an anion exchange column HiPrep Q XL 16/10 (GE Healthcare) to remove RNA contamination. The protein was bound to the column, washed with 2 column volumes of **low salt buffer** (**buffer D**) and eluted with a 10 column volumes-long gradient (0-100%) of **high salt buffer** (**buffer E**) (1 M NaCl, 30mM NH_4_Cl, 40 mM Tris:HCl pH 7.5, 5 mM MgCl_2,_  1 mM β-mercaptoethanol) while RNA remained captured on the column. The protein was polished by gel filtration in **HEPES:Polymix** buffer supplemented with 10% glycerol using HiLoad 16/600 Superdex 200 prep grade column (GE Healthcare), and concentrated on 50 MWCO centricons (Amicon) and the purity was confirmed by SDS-page. The protein was aliquoted and stored at -80°C.

***E. coli IF3***: For protein expression, the pET24a plasmid encoding 6His-tagged IF3 was transformed into *E. coli* BL21 (DE3) (Novagen). Transformants were selected on on LB-agar with 50 μg/mL kanamycin as a selectivity marker. Several colonies were inoculated in 50 mL of LB with kanamycin for overnight culturing at 37°C. The next day the overnight culture was diluted to OD_600_ 0.05 and grown at 37°C to OD_600_ 0.6, protein expression was induced with 1 mM IPTG (final concentration) and cells were grown for an additional 2 hours at 30°C. Cells were collected by centrifugation and frozen with liquid nitrogen.

For purification, cells were resuspended in **loading buffer** (350 mM NaCl, 20 mM Imidazole, 50 mM Tris:HCl pH 7.5, 5 mM MgCl_2_, 1 mM β-mercaptoethanol) supplemented with 0.1 mM PMSF protease inhibitor, 35 µg/ml lysozyme and 1 U/ml DNAse 1. Cells were lysed by a Stansted Fluid Power SPCH ultra high-pressure cell disrupter/homogenizer and cell debris was removed by centrifugation (40,000 rpm for 40 minutes). The supernatant was loaded onto a 1 mL HisTRAP HP (GE Healthcare) column pre-equilibrated with **loading buffer**. After sample loading, the column was washed with **high salt buffer (buffer B)** (2 M NaCl, 50 mM Tris:HCl pH 7.5, 20 mM imidazole, 5 mM MgCl_2_, 2 mM β-mercaptoethanol) and the protein was eluted with a gradient of **buffer supplemented with 0.5 M imidazole (buffer C)** (150 mM NaCl, 20 mM Tris:HCl pH 7.5, 5 mM MgCl_2_, 0.5 M imidazole, 1 mM β-mercaptoethanol). The eluted protein was collected, diluted in low salt buffer (**buffer D**) (30 mM NaCl, 30mM NH_4_Cl, 40 mM Tris:HCl pH 7.5, 5 mM MgCl_2,_  1 mM β-mercaptoethanol) and loaded onto a 5mL HiTrap SP cation exchange column (GE Healthcare) to remove RNA contamination. The protein was bound to the column and eluted with 60% **of high salt buffer** (**buffer E**) (1 M NaCl, 30mM NH_4_Cl, 40 mM Tris:HCl pH 7.5, 5 mM MgCl_2,_  1 mM β-mercaptoethanol) while RNA was eluted in flow-through. The protein was buffer exchanged to **HEPES:Polymix** buffer and concentrated on 50 MWCO centricons (Amicon) and the purity was confirmed by SDS-PAGE. The protein was aliquoted and stored at -80°C.

*Preparation* *of* E. faecalis *and* S. aureus *biomass*

*S. aureus* SH-1000 (Biosafety level 2) was kindly provided by Jan Oscarsson, Umeå University. The cultures were grown in 800 mL LB from OD_600_ 0.05 of diluted overnight culture untill late exponential phase, OD_600_ 2-2.5. Cells were collected by centrifugation 8000 rpm during 15 minutes at 4°C (TLA10.500 rotor, Beckman), washed with 20 mL media and collected in 50 mL falcon tubes by centrifugation in a bucket rotor (Eppendorf 5810 R). Collected cells were frozen as pellets in liquid nitrogen and stored at -80°C. Approximately 15 g of biomass was collected from in total 15 L of culture.

*E. faecalis* OG1RF was kindly provided by Dr. Barbara E. Murray (Health Science Center, University of Texas). The overnight culture was diluted to OD_600_ of 0.06, cells were grown in BHI at 37°C with shaking until OD_600_ reached 1.5 and collected by centrifugation at 5000 rcf during 15 minutes at +4°C. Cells were washed with cell opening buffer (20 mM Tris:HCl, 100 mM NH_4_Cl, 15 mM Mg(OAc)_2_, 0.5 mM EDTA, 3 mM mercaptoethanol, pH 7.4) and collected by centrifugation at 3,220 rcf during 20 minutes at +4°C. The biomass (15 g in total) was frozen in liquid nitrogen until further processing.

*Preparation* *of 70S ribosomes*

In the case of *S. aureus*, frozen cells were opened by cryomilling (Spex Freezer Mill) (8 cycles at 14 fps frequency interspersed with 2 minute work-rest intervals) and the powder was melted on ice during 3 hours before opening the tube. After melting, the tube with cells was opened in a class II biosafety cabinet (ESCO), 50 mL of cell opening buffer (100 mM NH_4_Cl, 15 mM MgAc_2_, 0.5 mM EDTA, 3 mM β-mercaptoethanol, 20 mM Tris:HCl pH 7.5), supplemented with 0.4 mU Turbo DNAse (Thermo Fisher Scientific), 0.1 mM PMSF and 35 μg/ml lysozyme and lysate was incubated on ice for one hour. In the case of *E. faecalis,* cells were resuspended in the same opening buffer and were opened using the Stansted Fluid Power SPCH ultra high-pressure cell disrupter/homogenizer at 350 MPa. The suspension was passed through 3 times.

Lysed cells were clarified by centrifugation for 40 minutes at 40,000 rpm (Ti 45 rotor, Beckman). The supernatant was divided between 6 tubes (13 mL per tube), loaded onto a 40 mL **sucrose cushion** (1.1 M sucrose, 500 mM NH_4_Cl, 15 mM MgAc_2_, 0.5 mM EDTA, 3 mM β-mercaptoethanol, 20 mM Tris:HCl pH 7.5) and centrifuged for 18-19 hours at 28,000 rpm. The ribosomal pellet was dissolved in **high salt buffer** (500 mM NH_4_Cl, 15 mM MgAc_2_, 0.5 mM EDTA, 3 mM β-mercaptoethanol, 20 mM Tris:HCl pH 7.5 supplemented with 0.5-1 mM puromycin) and incubated for 1 hour at 4°C with gentle mixing on an orbital shaker. 12 ml of the resuspended pellet was loaded onto a 40 mL **sucrose cushion** in 2 tubes and centrifugation was repeated for either for 8 hours (35,000 rpm) or for 19 hours (28,000 rpm) Ribosomal pellets were resuspended in 15 ml of 1x **overlay buffer** (60 mM NH_4_Cl, 15 mM MgAc_2_, 0.25 mM EDTA, 3 mM β-mercaptoethanol, 20 mM Tris:HCl pH 7.5). Subunits and 70S ribosomes were separated by a gradient of 10-40% sucrose solution in **overlay buffer** in a zonal rotor (Ti 15, Beckman) during 17 hours at 21,000 rpm. The peak containing 70S ribosomes was collected, and the ribosomes were pelleted by centrifugation for 20 hours at 35,000 rpm. The pellet was dissolved in 1 ml of **HEPES:Polymix buffer** (20 mM HEPES:KOH pH 7.5, 2 mM DTT, 5 mM MgOAc_2_, 95 mM KCl, 5 mM NH_4_Cl, 0.5 mM CaCl_2_, 8 mM putrescine, 1 mM spermidine). Ribosome concentration was measured by absorbance at 260 nm, 1 OD_260_ corresponds to 23 nM of 70S ribosomes. The quality of 16S and 23S rRNA was checked by electrophoresis in a 1% agarose gel. The obtained 70S ribosomes were aliquoted per 50 μL in PCR tubes, frozen in liquid nitrogen and stored at -80°C.

*Preparation of 70S initiation complexes (70S IC)*

Initiation complexes (ICs) were prepared by combining 70S ribosomes (final concentration of 4 μM) with IF2 (2 μM), IF1 (1.5 μM), IF3 (1.5 μM), ^35^S-fMet-tRNA^Met^ (6 μM), mRNA MF (6 μM, 5'-GGCAAGGAGGUAAAAAUGUUCAAA-3'), 1 mM GTP and 2 mM DTT **HEPES:Polymix buffer**. The reaction mix was incubated at 37°C for 30 minutes and initiation factors and unbound RNAs were removed by pelleting ICs through a sucrose cushion (1.1 M sucrose, **polymix buffer** 15 mM MgCl_2_) at 50,000 rpm during 2 hours (TLS-55, Beckman). The supernatant was aspirated and the pellet was dissolved in 100 μL of **HEPES:Polymix buffer**, 5 mM MgCl_2_. ^35^S-fMet-tRNA^Met^ occupancy on ribosomes was measured as the fraction of ^35^S-fMet-tRNA^Met^ concentration (calculated by scintillation counting) and ribosome concentration (measured spectrophotometrically, 1 OD_260_ nm corresponds to 23 nmole of 70S). The occupancy was 60-80% for *S. aureus* ICs and around 45% for *E. faecalis* ICs. This is a relatively low percentage as compared to the ~95% for *E. coli* ICs prepared from the MRE600 strain that lacks RNAse I and is therefore well-suited for preparation of active ribosomes (6,7). The wild type *S. aureus* SH-1000 and *E. faecalis* OG1RF strains were not optimised for purification of ribosomes, and likely contain a large concentration of nucleases and proteases reducing the functional activity of purified 70S ribosomes. 70S ICs were aliquoted per 20 μl in PCR tubes, frozen in liquid nitrogen and stored at -80°C.

*Puromycin reaction assay*

The puromycin reaction was carried out at 37°C in **HEPES:Polymix buffer** (1). All antibiotics and nucleotide solutions were prepared in HEPES:Polymix buffer, pH adjusted to 7.5. After 5 minutes of preincubation of 70S ICs (0.5 μM final concentration) with all the reaction components (nucleotides, antibiotics and ABCF ARE) the zero time point was taken, and immediately after that puromycin was added to a final concentration of 1 mM. Each time point (4-6 μL of reaction, i.e. 2-3 pmol of ^35^S-Met-labelled IC corresponds to 1,000-2,000 CPM) was transferred to 95 μL of 10% TCA supplemented with 15 μg of bovine serum albumin (BSA) used as a coprecipitant to visualize the pellet, and incubated on ice for at least 5 minutes. After that the samples were spun down 20 minutes at 20,000 rcf, and the 100 μL supernatant transferred into a new Eppendorf tube containing 400 μL of 1 M Tris:HCl pH 8.0. This step is necessary in order to neutralize the pH since low pH decreases the scintillation signal by an order of magnitude. The precipitate was dissolved in 500 μL of 1 M Tris:HCl pH 8.0 for 30 minutes at 65°C with shaking. Finally, 500 μL of samples were transferred to scintillation vials containing 5 mL of ScintiSafe 3 scintillation cocktail (FisherScientific), mixed carefully and counted on a TRI-CARB 4910TR 110 V scintillation counter (PerkinElmer). The percentage of ^35^S-fMethione released from the 70S IC was calculated by dividing the signal from the supernatant by the sum of signals from supernatant and pellet. The total sum of the CPMs was similar for all the time points (7257±1306 CPM, i.e. ±18 %). The data were normalized to zero value using the zero point from each experiment and to maximum value (100%) using the maximal signal obtained from non-inhibited puromycin reaction, and plotted in IGOR Pro (WaveMetrics).

**
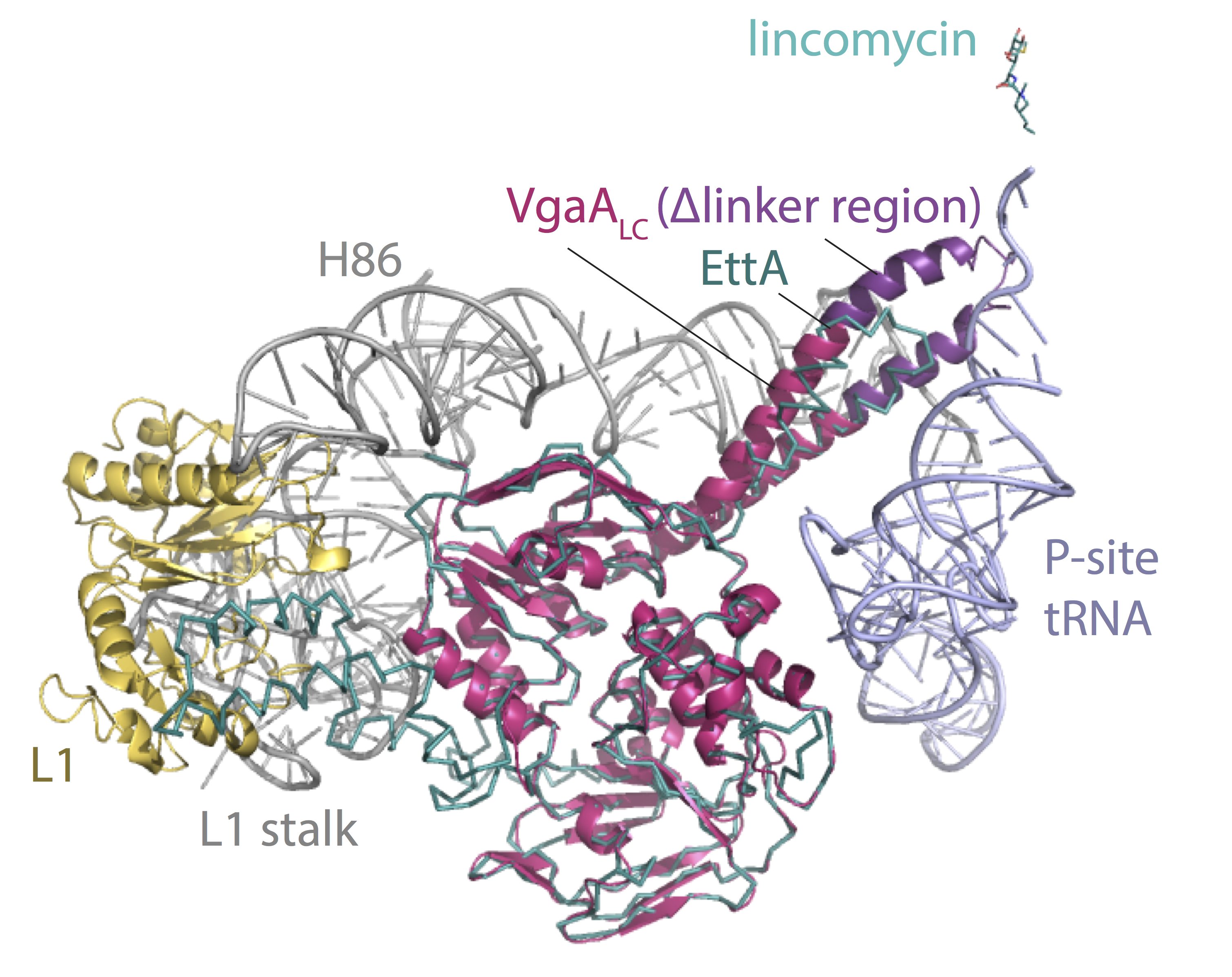
Supplementary Figure 1 | Structural alignment of ribosome-bound *S. aureus* VgaA_LC_ homology model and *E. coli* EttA.** Swiss Model (8) was used to build a homology model of VgaA_LC_, using EttA (PDB ID 3J5S) as the template. As the VgaA_LC_ linker could not be modeled due to lack of homology, QUARK (9) was used for *ab initio* structure modeling of this region, and the resulting structure was aligned back to the homology model using the structural alignment method of MacPyMOL (10). The tip of the linker that is deleted in VgaA_LC_∆L is shaded in purple. The relative location of lincomycin was found by during a structural alignment of the ribosomal components of 3J5S with the structure of lincomycin on the large ribosomal subunit, PDB ID 5HKV (11).


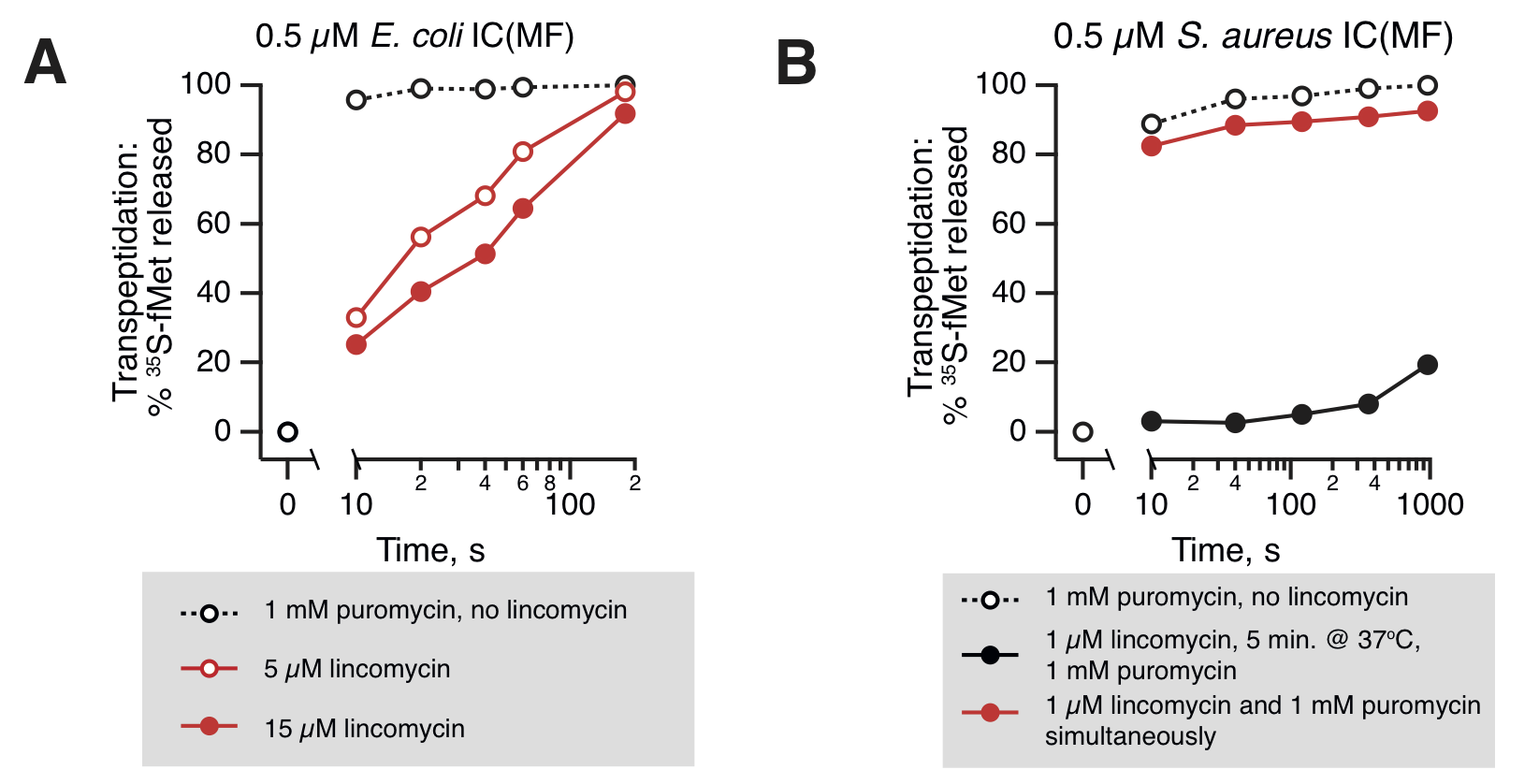


**Supplementary Figure 2 | Competition of puromycin and PTC blocking antibiotics for *E. coli* and *S. aureus* 70S IC.**

At the concentration of 5 (empty red circles) and 15 (filled red circles) µM lincomycin slows down ^35^S-Methionine release from P-site of *E. coli* 70S ribosomal initiation complexes (70S IC) by 1 mM puromycin, but fails to abrogate the release completely (**A**). Lincomycin was preincubated with *E. coli* 70S IC 37°C for 5 minutes prior to the addition of puromycin to final concentration of 1 mM. Puromycin kinetically outcompetes the PTC inhibitor lincomycin on *S. aureus* 70S, showing the validity of the assay in our conditions (**B**). When added to *S. aureus* 70S IC simultaneously with 1 µM lincomycin, 1 mM puromycin efficiently outcompetes the antibiotic (filled red circles). When 1 µM lincomycin is preincubated with IC 37°C for 5 minutes prior to the addition of puromycin, lincomycin efficiently abrogated the puromycin reactivity (filled black circles). 0.5 µM *E. coli* (**A**) or *S. aureus* (**B**) 70S IC(MF) were programmed with *E. coli* ^35^S-fMet-tRNA_i_^fMet^ and synthetic mRNA(MF). The experiments were performed at 37°C in HEPES:Polymix pH 7.5 buffer, 5 mM Mg^2+^.


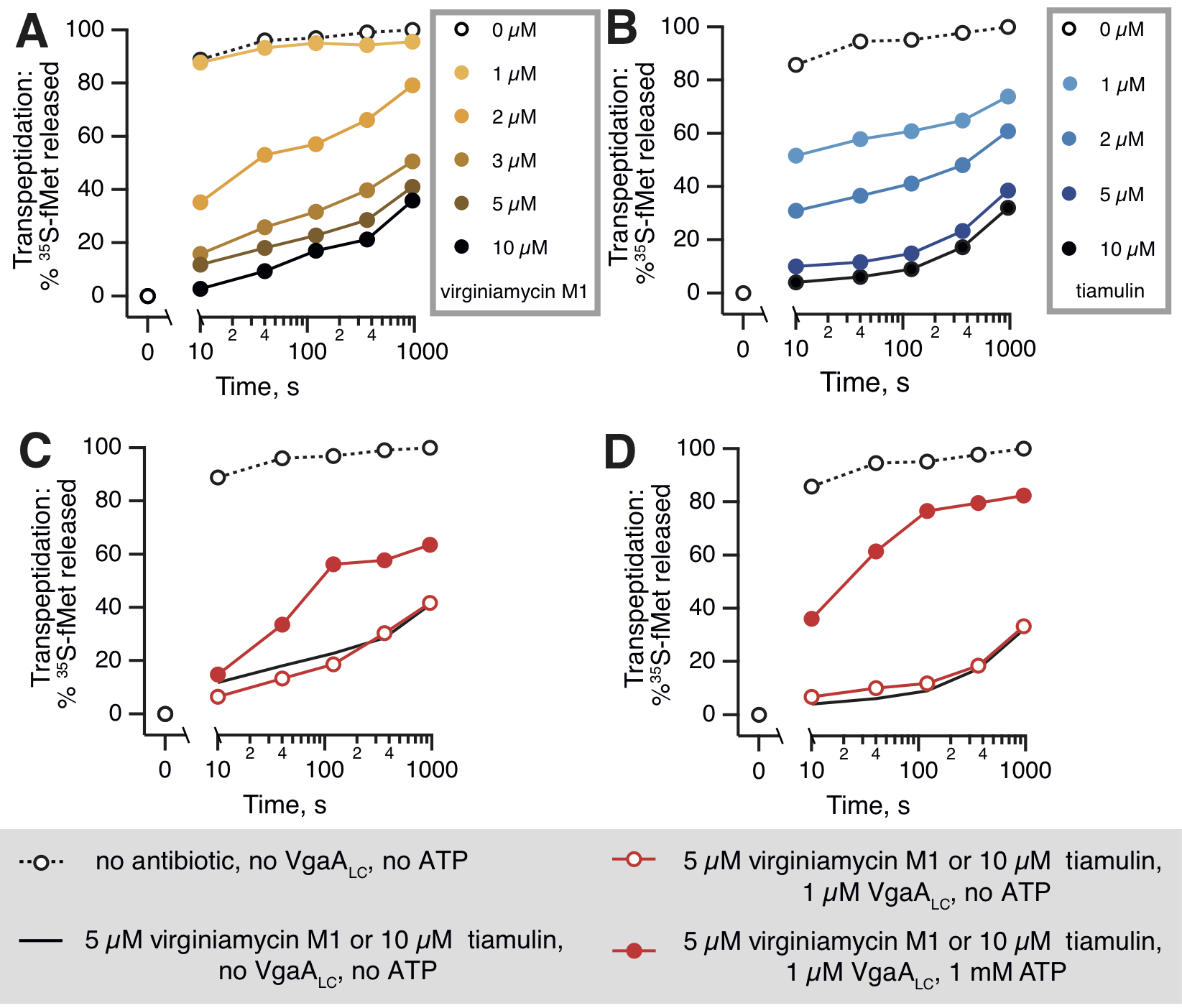


**Supplementary Figure 3 | *S. haemolyticus*** **VgaA_LC_ ATP-dependently rescues puromycin reactivity of *S. aureus* 70S IC(MF) inhibited by virginiamycin M1 or tiamulin.** Increasing concentrations of virginiamycin M1 inhibit the ^35^S-Methionine release by 1 mM puromycin but fail to abrogate it completely (**A**). Pleuromutilin tiamulin abrogates puromycin reactivity at 10 µM (**B**). In the presence of 1 mM ATP (filled red circles) 1 µM *S. haemolyticus* VgaA_LC_ rescues the puromycin reactivity inhibited by 5 µM virginiamycin M1 (**C**) or 10 µM tiamulin (**D**). In the absence of nucleotides (empty red circles) or in the presence of 1 mM ADP (filled brown circles) addition of 1 µM VgaA_LC_ has no effect. The experiments were performed at 37°C in HEPES:Polymix pH 7.5 buffer, 5 mM Mg^2+^. 0.5 µM *S. aureus* 70S IC(MF) were programmed with *E. coli* ^35^S-fMet-tRNA_i_^fMet^ and synthetic mRNA(MF).


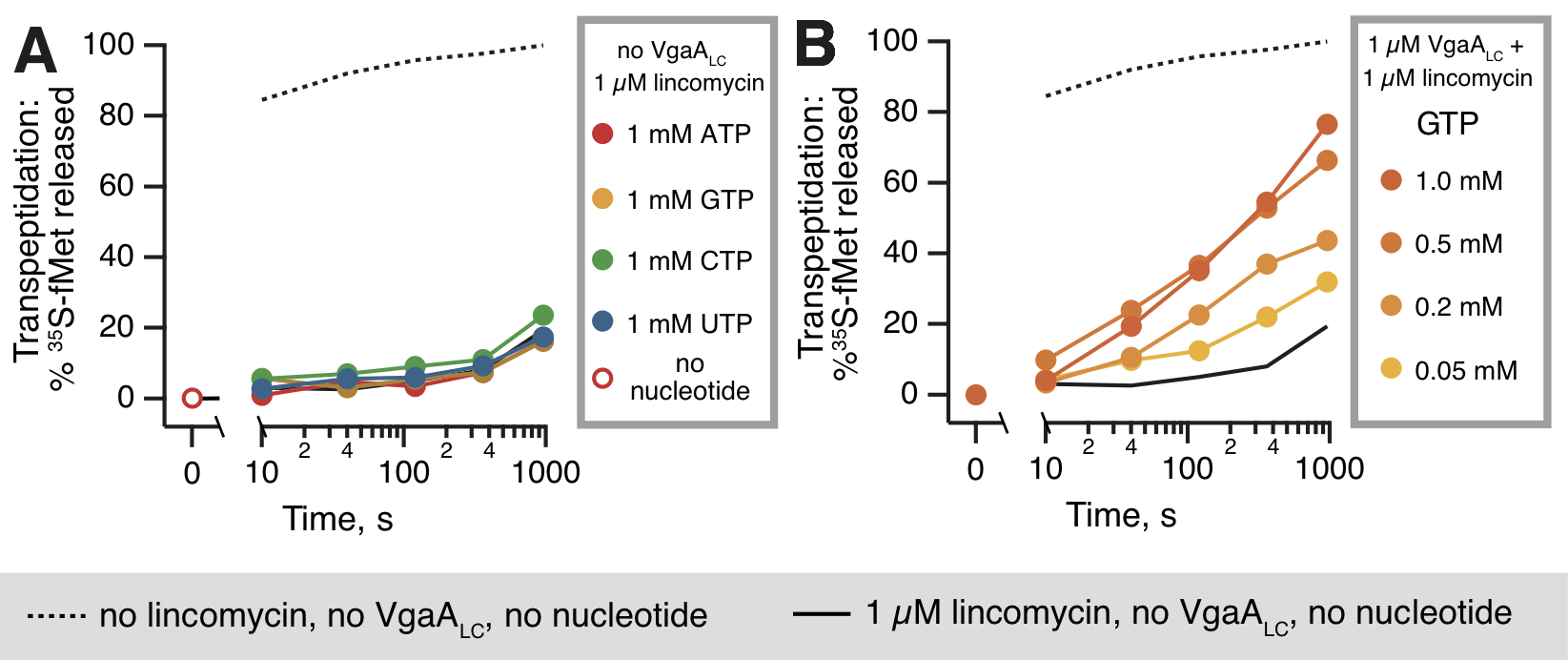


**Supplementary Figure 4 | Specificity of the NTP’s effect on VgaA_LC_-mediated lincomycin resistance.** In the absence of VgaA_LC_ addition of either 1 mM ATP, GTP, CTP or UTP has no effect on the puromycin reactivity of *S. aureus* 70S IC(MF) inhibited by 1 µM lincomycin (**A**). Puromycin reactivity is progressively rescued by 1 µM VgaA_LC_ and increasing concentrations of GTP (**B**). The puromycin reactivity saturates at 0.5 mM GTP. The experiments were performed at 37°C in HEPES:Polymix pH 7.5 buffer, 5 mM Mg^2+^. 0.5 µM *S. aureus* 70S IC(MF) were programmed with *E. coli* ^35^S-fMet-tRNA_i_^fMet^ and synthetic mRNA(MF).


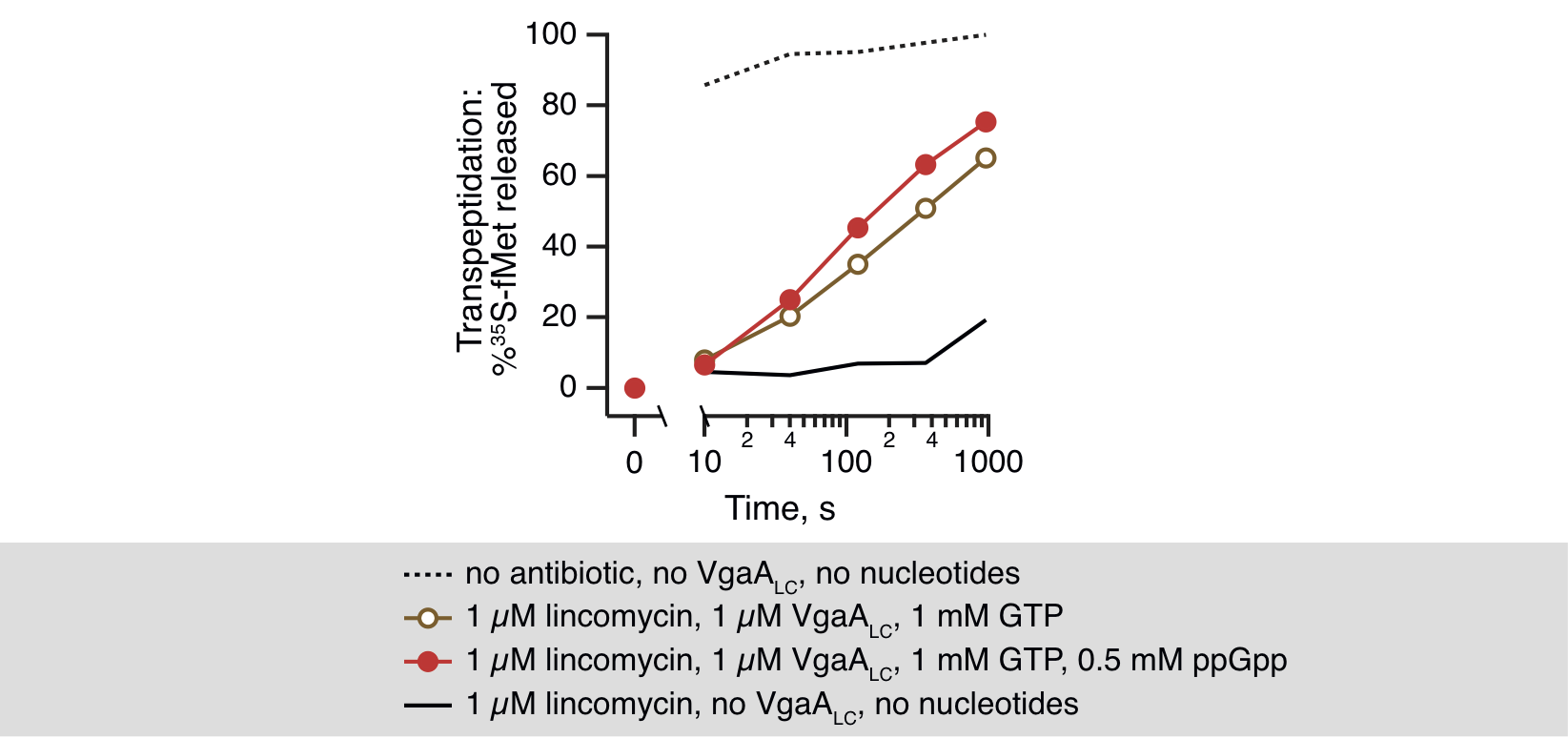


**Supplementary Figure 5 | ppGpp does not inhibit VgaA_LC_-mediated lincomycin resistance.** Addition of either 0.5 mM ppGpp has no significance effect on the VgaA_LC_-mediated rescue of the puromycin reactivity of *S. aureus* 70S IC(MF) inhibited by 1 µM lincomycin. The experiments were performed at 37°C in HEPES:Polymix pH 7.5 buffer, 5 mM Mg^2+^. 0.5 µM *S. aureus* 70S IC(MF) were programmed with *E. coli* ^35^S-fMet-tRNA_i_^fMet^ and synthetic mRNA(MF).

**Supplementary references:**

1. Jelenc, P.C. and Kurland, C.G. (1979) Nucleoside triphosphate regeneration decreases the frequency of translation errors. *Proceedings of the National Academy of Sciences of the United States of America*, **76**, 3174-3178.

2. Antoun, A., Pavlov, M.Y., Tenson, T. and Ehrenberg, M.M. (2004) Ribosome formation from subunits studied by stopped-flow and Rayleigh light scattering. *Biol Proced Online*, **6**, 35-54.

3. Andréasson, C., Fiaux, J., Rampelt, H., Mayer, M.P. and Bukau, B. (2008) Hsp110 is a nucleotide-activated exchange factor for Hsp70. *J Biol Chem*, **283**, 8877-8884.

4. Novotna, G. and Janata, J. (2006) A new evolutionary variant of the streptogramin A resistance protein, Vga(A)LC, from Staphylococcus haemolyticus with shifted substrate specificity towards lincosamides. *Antimicrob Agents Chemother*, **50**, 4070-4076.

5. Singh, K.V., Weinstock, G.M. and Murray, B.E. (2002) An Enterococcus faecalis ABC homologue (Lsa) is required for the resistance of this species to clindamycin and quinupristin-dalfopristin. *Antimicrob Agents Chemother*, **46**, 1845-1850.

6. Kurylo, C.M., Alexander, N., Dass, R.A., Parks, M.M., Altman, R.A., Vincent, C.T., Mason, C.E. and Blanchard, S.C. (2016) Genome Sequence and Analysis of Escherichia coli MRE600, a Colicinogenic, Nonmotile Strain that Lacks RNase I and the Type I Methyltransferase, EcoKI. *Genome Biol Evol*, **8**, 742-752.

7. Cammack, K.A. and Wade, H.E. (1965) The sedimentation behaviour of ribonuclease-active and -inactive ribosomes from bacteria. *Biochem J*, **96**, 671-680.

8. Biasini, M., Bienert, S., Waterhouse, A., Arnold, K., Studer, G., Schmidt, T., Kiefer, F., Gallo Cassarino, T., Bertoni, M., Bordoli, L. *et al.* (2014) SWISS-MODEL: modelling protein tertiary and quaternary structure using evolutionary information. *Nucleic Acids Res*, **42**, W252-258.

9. Xu, D. and Zhang, Y. (2012) Ab initio protein structure assembly using continuous structure fragments and optimized knowledge-based force field. *Proteins*, **80**, 1715-1735.

10. Schrodinger, L. (2015) The PyMOL Molecular Graphics System, Version 1.8.

11. Matzov, D., Eyal, Z., Benhamou, R.I., Shalev-Benami, M., Halfon, Y., Krupkin, M., Zimmerman, E., Rozenberg, H., Bashan, A., Fridman, M. *et al.* (2017) Structural insights of lincosamides targeting the ribosome of Staphylococcus aureus. *Nucleic Acids Res*, **45**, 10284-10292.
